# High ambient temperature in pregnancy and risk of childhood acute lymphoblastic leukaemia: an observational study

**DOI:** 10.1016/S2542-5196(24)00121-9

**Published:** 2024-07

**Authors:** Tormod Rogne, Rong Wang, Pin Wang, Nicole C Deziel, Catherine Metayer, Joseph L Wiemels, Kai Chen, Joshua L Warren, Xiaomei Ma

**Affiliations:** Department of Chronic Disease Epidemiology, Yale School of Public Health, New Haven, CT, USA; Center for Perinatal, Pediatric and Environmental Epidemiology, Yale School of Public Health, New Haven, CT, USA; Department of Chronic Disease Epidemiology, Yale School of Public Health, New Haven, CT, USA; Department of Environmental Health Sciences, Yale School of Public Health, New Haven, CT, USA; Department of Environmental Health Sciences, Yale School of Public Health, New Haven, CT, USA; School of Public Health, University of California, Berkeley, CA, USA; Center for Genetic Epidemiology, Department of Population and Public Health Sciences, Keck School of Medicine, University of Southern California, Los Angeles, CA, USA; Department of Environmental Health Sciences, Yale School of Public Health, New Haven, CT, USA; Department of Biostatistics, Yale School of Public Health, New Haven, CT, USA; Department of Chronic Disease Epidemiology, Yale School of Public Health, New Haven, CT, USA

## Abstract

**Background:**

High ambient temperature is increasingly common due to climate change and is associated with risk of adverse pregnancy outcomes. Acute lymphoblastic leukaemia is the most common malignancy in children, the incidence is increasing, and in the USA disproportionately affects Latino children. We aimed to investigate the potential association between high ambient temperature in pregnancy and risk of childhood acute lymphoblastic leukaemia.

**Methods:**

We used data from California birth records (children born from Jan 1, 1982, to Dec 31, 2015) and California Cancer Registry (those diagnosed with childhood cancer in California from Jan 1, 1988, to Dec 31, 2015) to identify acute lymphoblastic leukaemia cases diagnosed in infants and children aged 14 years and younger and controls matched by sex, race, ethnicity, and date of last menstrual period. Ambient temperatures were estimated on a 1-km grid. The association between ambient temperature and acute lymphoblastic leukaemia was evaluated per gestational week, restricted to May–September, adjusting for confounders. Bayesian meta-regression was applied to identify critical exposure windows. For sensitivity analyses, we evaluated a 90-day pre-pregnancy period (assuming no direct effect before pregnancy), adjusted for relative humidity and particulate matter less than 2·5 microns in aerodynamic diameter, and constructed an alternatively matched dataset for exposure contrast by seasonality.

**Findings:**

6849 cases of childhood acute lymphoblastic leukaemia were identified and, of these, 6258 had sufficient data for study inclusion. We also included 307 579 matched controls. Most of the study population were male (174 693 [55·7%] of the 313 837 included in the study) and of Latino ethnicity (174 906 [55·7%]). The peak association between ambient temperature and risk of acute lymphoblastic leukaemia was observed in gestational week 8, where a 5°C increase was associated with an odds ratio of 1·07 (95% CI 1·04–1·11). A slightly larger effect was seen among Latino children (OR 1·09 [95% CI 1·04–1·14]) than non-Latino White children (OR 1·05 [1·00–1·11]). The sensitivity analyses supported the results of the main analysis.

**Interpretation:**

Our findings suggest an association between high ambient temperature in early pregnancy and risk of childhood acute lymphoblastic leukaemia. Further replication and investigation of mechanistic pathways might inform mitigation strategies.

**Funding:**

Yale Center on Climate Change and Health, The National Center for Advancing Translational Science, National Institutes of Health.

## Introduction

Climate change in the form of high ambient temperature has had devastating consequences for human health worldwide and is projected to have an even greater effect in the future.^1^ Racial and ethnic minority groups experience a disproportionate burden of heat exposure.^1-3^ It is increasingly clear that high ambient temperature during pregnancy has negative effects on birth outcomes, especially in racial and ethnic minority groups.^4^ However, very little is known about the long-term outcomes for infants because of exposure to high ambient temperature during pregnancy.

Childhood acute lymphoblastic leukaemia is an important potential outcome. Acute lymphoblastic leukaemia is the most common childhood malignancy with more than 50 000 new cases diagnosed every year worldwide, and the incidence is steadily increasing.^5^ In the USA, there is considerable disparity by race and ethnicity, where Latino children have about a 40% higher risk of developing acute lymphoblastic leukaemia than do non-Latino White children.^5^ This difference in risk is not explained by genetic differences.^6^

Childhood acute lymphoblastic leukaemia has a prenatal origin, and most children with the disease have pre-leukaemic clones at the time of birth.^7,8^ Exposure during the first trimester is suspected to be the most critical because this is when the most profound developmental alterations in haematopoiesis occur (lymphopoiesis starts at about gestational week 8).^9^ Environmental exposures occurring during pregnancy such as air pollution (particularly in early pregnancy) have been associated with an increased risk of childhood acute lymphoblastic leukaemia.^10-12^ The disease has been observed more frequently among those born in late winter, aligning with high ambient temperature in early pregnancy.^13,14^ There are multiple reasons to suspect that maternal exposure to high ambient temperature might initiate the pathogenesis of acute lymphoblastic leukaemia in fetal life. First, pre-leukaemic clones might be caused by oxidative stress,^7^ and high ambient temperature increases oxidative stress.^15-17^ Second, epigenetic changes, inflammatory markers, heat shock proteins, and stress hormones increase in response to heat,^16,17^ which in turn has been linked to an increased risk of childhood acute lymphoblastic leukaemia.^18-20^

Using a population-based linkage study derived from the general birth cohort in California (1982–2015), we conducted a nested case-control study to evaluate the potential association between high ambient temperature during pregnancy and risk of acute lymphoblastic leukaemia in infants. We aimed to identify critical windows of exposure and to evaluate possible differences by racial and ethnic groups. Our hypothesis was that any harmful effect of high ambient temperature would be greatest during the first trimester and that Latino children would be more affected by high temperatures than non-Latino children. To our knowledge, this is the first study to directly evaluate the association between ambient temperature in pregnancy and the risk of cancer in infants.

## Methods

### Study design and population

This study leveraged the California Linkage Study of Early-Onset Cancers, which is a population-based, statewide linkage study that included children born in California from Jan 1, 1982, to Dec 31, 2015, and diagnosed with childhood cancer in California from Jan 1, 1988, to Dec 31, 2015.^10,21^ Non-cancer controls were selected from the statewide birth records. Information on cancer status was gathered from the California Cancer Registry, and birth records from the Center for Health Statistics and Informatics, both within the California Department of Public Health. This study received approval from the institutional review boards at California Department of Public Health, Yale University, University of California, Berkeley, and University of Southern California.

We used the International Classification of Diseases for Oncology (3rd edition) to identify cases, for which codes of 9811–18, 9826, and 9835–37 were considered consistent with a diagnosis of acute lymphoblastic leukaemia. An age-limit of 14 years or younger was used to define childhood acute lymphoblastic leukaemia. We initially identified cases of childhood acute lymphoblastic leukaemia, of whom had sufficient data to be included in the current study. We excluded children who had missing residential address at the time of birth; had mothers who resided outside of California; had missing information on gestational age, birthweight, birth order, mother’s birthplace, or delivery mode; had congenital malformations, including Down syndrome, or had missing information on this item; or had no available controls (appendix p 6). We next selected non-cancer controls per case matched on sex, race, and ethnicity (which are associated with the risk of childhood acute lymphoblastic leukaemia),^5^ and date of mother’s last menstrual period (plus or minus 7 days) to ensure that each case and their matched controls were at the same stage of pregnancy at the same time.

### Procedures

Maternal residential addresses were based on street addresses for those born from Jan 1, 1997, to Dec 31, 2015, and on zip codes for those born from Jan 1, 1982, to Dec 31, 1996, and were geocoded at the greatest available spatial precision.^21^ For temperature ascertainment, we obtained daily maximum and minimum 2 m air temperature readings at a spatial resolution of 1 km from the Daily Surface Weather Data on a 1-km Grid for North America, version 4 (Daymet), as modelled by Thornton and colleagues (appendix p 2).^22^ The exposure of interest was daily mean temperature which was estimated as the average of daily minimum and maximum temperatures. Water vapour pressure was also acquired from Daymet, and relative humidity was computed at the same resolution using both water vapour pressure and daily mean temperature.^23^

Regarding air pollution, daily particulate matter less than 2·5 microns in aerodynamic diameter (PM_2·5_) was estimated at a 1-km resolution based on a validated chemical transport model with covariates including satellite-derived aerosol optical depth, weather patterns, population density, and land use, as previously described.^10^ Weekly averages were calculated based on daily estimates. These data were available for the period between Jan 1, 2000, and Dec 31, 2015.

Data on several other variables were retrieved to describe our population and account for potential confounding. Birth records were used to gather the following information: Gestational age at birth (in days, based on the last menstrual period method), date of birth, race and ethnicity (Latino, non-Latino White, non-Latino Black, non-Latino Asian or Pacific Islander, other), sex (male or female), birth order (first, second, third), maternal age (<20, 20–24, 25–29, 30–34, ≥35 years), paternal age (<25, 25–29, 30–34, 35–39, ≥40 years), number of years of maternal education (<8, 9–11, 12, 13–15, ≥16 years), birthweight (g), and mode of delivery (vaginal or caesarean section). Maternal residential addresses were linked to 2000 Census block group data to obtain the Social Deprivation Index (tertiles based on the distribution among controls).^24^ This index considers seven demographic characteristics including poverty, level of education, and housing.^24^ Date of last menstrual period was estimated based on date of birth and registered gestational age at birth.

### Statistical analysis

We evaluated the association between weekly mean ambient temperature (from daily means) and the risk of childhood acute lymphoblastic leukaemia 1 week at a time before running Bayesian meta-regression analyses (statistical model specified below). In sensitivity analyses, the study period was restricted to the years with available street addresses (1997 and onwards), and weekly temperatures were averaged based on daily minimum and daily maximum temperatures. All results are expressed as odds ratio (ORs) for acute lymphoblastic leukaemia per 5°C increase in mean weekly ambient temperature, unless otherwise specified. Given that we wanted to evaluate the effect of high ambient temperature, we restricted the season of interest to the warm period May 1–Sept 30 each year. For any given week of exposure, at least 1 day of that week had to be in the warm period (appendix p 8). The warm season represents roughly 42% of the full year, so for any given week, the sample size was roughly 42% of the total study population (appendix p 5).

To identify critical windows of exposure, we analysed our data in two stages. First, we ran conditional logistic regression models, separately for each week, for which the association between mean ambient temperature and acute lymphoblastic leukaemia risk was estimated, along with a standard error for the estimate. Next, we used a Bayesian meta-regression framework, for which the estimates and standard errors from the first stage were used as input for the second analysis stage. For the second analysis stage, we extended the original critical window variable selection method of Warren and colleagues to the meta-regression setting (appendix pp 2-4).^25^

In addition to evaluating in-pregnancy associations, we evaluated the 13 weeks (90 days) preceding last menstrual period as an approximate negative control exposure period.^26^ As embryonic lymphopoiesis starts around gestational week 8,^9^ we hypothesised that we would see no association between ambient temperature and acute lymphoblastic leukaemia in the weeks preceding pregnancy.

In our conditional logistic regression analyses matched on race, ethnicity, sex, and date of last menstrual period (plus or minus 7 days), we adjusted for potential confounders identified through literature search and construction of directed acyclic graphs: birth order, maternal and paternal age, maternal education, Social Deprivation Index, seasonality and time trend (accounted for by matching on last menstrual period), and residential address at the time of birth (accounted for by matching on residential address; appendix p 9). We did not include the matching factors as covariates as this is not required in conditional logistic regression when matching is exact.^27^ The exposure to ambient temperature was measured at the population level therefore it was appropriate to adjust for population-level covariates such as the Social Deprivation Index.^28^ Given that exposure contrast to ambient temperature is achieved by time or space, we could not simultaneously match on date of last menstrual period and residential address. Instead, we considered date of last menstrual period to be the more important confounder of the two, which was considered in the primary analyses, while residential address was accounted for in a sensitivity analysis of a secondary matched dataset. Unless stated otherwise, all characteristics and analyses presented were based on the primary matched dataset. We considered pregnancy complications and birth outcomes to be potential mediators of the association between high ambient temperature in pregnancy and risk of acute lymphoblastic leukaemia and therefore they were not adjusted for.

After identifying the gestational week during which the association between ambient temperature and risk of acute lymphoblastic leukaemia was the greatest (ie, the largest estimated association that was significant), we used this gestational week as the basis for subsequent prespecified subgroup analyses. First, we conducted analyses separately for Latino and non-Latino White subjects; the other racial or ethnic groups had too few cases for stratified analyses. The other subgroup analysis was stratified on age of diagnosis. Subgroup differences were compared by calculating the z-statistic.^29^ Finally, as the critical windows of exposure might have differed by subgroup, we also conducted post-hoc week-specific analyses from pre-pregnancy through to the end of pregnancy for the mentioned subgroups.

We conducted sensitivity analyses to evaluate whether the main findings were affected by humidity levels or PM_2·5_ by adding them as covariates. Air pollution is generally considered a mediator of the association between high ambient temperature and adverse health outcomes.^15^ If we observed a reduction in the magnitude of the association between temperature and acute lymphoblastic leukaemia after adding PM_2·5_ to the model, we would proceed with formal mediation analyses. In the week identified to have the largest estimated association between ambient temperature and acute lymphoblastic leukaemia risk, we also conducted analyses within tertiles of PM_2·5_ to assess whether the magnitude of the association differed by levels of PM_2·5_.

We conducted a regression analysis that included mean ambient temperature modelled using a restricted cubic spline with 5 knots (5th, 27·5th, 50th, 72·5th and 95th percentiles), adjusting for the same confounders as the main analysis. The non-linear analysis was only carried out in the gestational week with the largest estimated association with ambient temperature exposure that was significant. A log-likelihood ratio test was run to evaluate whether the non-linear model was a better fit than the linear model.

To address potential confounding due to residential address at birth, we created a secondary matched dataset where we matched cases and controls on year of last menstrual period, sex, race, ethnicity, and residential address at birth within 10 km. Thus, the difference between the main dataset and the secondary matched dataset was that the former compared pregnancies occurring at the same time (plus or minus 7 days) but at different locations, while the secondary matched dataset compared pregnancies occurring in the same location (within 10 km) but at different times during the same year (appendix p 10). This analysis was not restricted to the warm period but was otherwise adjusted for the same confounders as in the other analyses. Because of seasonal variations in air pollution levels, we conducted a sensitivity analysis where we included PM_2·5_ as a covariate.^30^ Due to the strict matching criteria, the secondary matched dataset included four controls per case, and not all cases had available controls. Our hypothesis was that the secondary matched dataset would reveal a similar positive association between ambient temperature in the first trimester and risk of acute lymphoblastic leukaemia. Given that this dataset was not restricted to the warm season, and as it was matched on year and location but not date of last menstrual period, we expected to see an artificial inverse association about 6 months after the peak positive association (ie, in the third trimester). An increased proportion of cases (compared with controls) conceived in early summer would result in higher mean ambient temperature (compared with controls) in early gestational weeks, but 6 months later, a greater proportion of cases (compared with controls) would be in the final weeks of pregnancy during the winter months.

The two-stage meta-regression analyses used the rjags package (version 4.13) in R (version 4.2.2), and all other analyses were performed with SAS version 9.4, with a statistical significance level of 0·05.

### Role of the funding source

The funders of the study had no role in data collection, data analysis, data interpretation, or writing of the report.

## Results

For the warm season in the years 1982–2015, the mean of the weekly mean ambient temperature was 21·5°C (SD 3·7; IQR 19·0–23·9), and the lowest and highest recorded weekly mean temperatures were 0·0°C and 40·1°C, respectively. Mean weekly temperatures by cases and controls are presented in the appendix (p 11).

We initially identified 6849 cases of childhood acute lymphoblastic leukaemia, of which 6258 had sufficient data to be included in the current study (appendix p 6). We next selected 50 (or as many available who met our criteria) non-cancer controls, which resulted in a total of 307 579 controls. The distribution of the distance between cases and matched controls are presented in the appendix (p 7).

Most of the study population were male (174 693 [55·7%] of the 313 837 included in the study population) and of Latino ethnicity (174 906 [55·7%]; table). Sex, race, ethnicity, and year of birth were equally distributed between cases and controls as per matching design. Compared with controls, cases had slightly greater birthweight and were more likely to have mothers with higher education. Among the 6258 cases, 3636 (58·1%) were diagnosed before age 5 years, 1769 (28·3%) at the age of 5–9 years, and 853 (13·6%) between ages 10 and 14 years.

**Figure 1:**
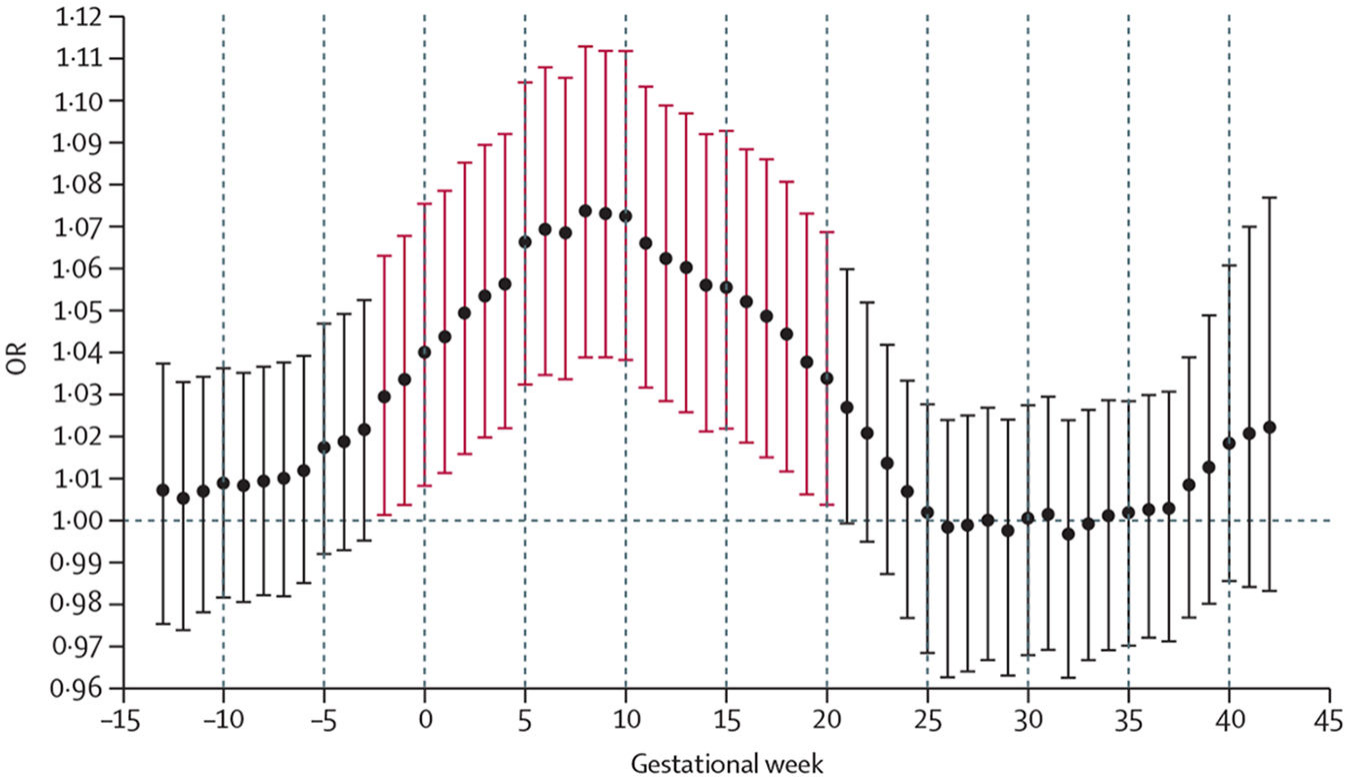
Gestational week-specific associations between high ambient temperature and risk of childhood acute lymphoblastic leukaemia Results from the two-stage Bayesian meta-regression analysis of ambient temperature and risk of childhood acute lymphoblastic leukaemia. Accounted for race, ethnicity, birth order, maternal and paternal age, maternal education, Social Deprivation Index, date of last menstrual period plus or minus 7 days (ie, seasonality and time trend), and infant sex. Unit of exposure per 5°C increase in mean weekly ambient temperature. Vertical bars represent 95% CIs. Significant associations between high ambient temperature and childhood acute lymphoblastic leukaemia are shown in red. OR=odds ratio.

**Figure 2:**
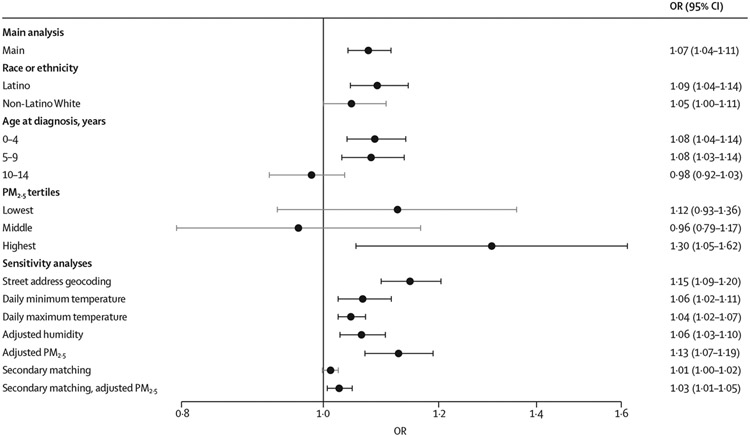
Subgroup and sensitivity analyses of ambient temperature in gestational week 8 and risk of childhood acute lymphoblastic leukaemia Results from the two-stage Bayesian meta-regression analysis of ambient temperature and risk of childhood acute lymphoblastic leukaemia in gestational week 8 (identified as most susceptible week of exposure) in subgroup and sensitivity analyses. All analyses accounted for race, ethnicity, birth order, maternal and paternal age, maternal education, Social Deprivation Index, date of last menstrual period plus or minus 7 days (ie, seasonality and time trend), and infant sex, with the following exceptions: the sensitivity analyses with humidity and PM_2·5_ additionally adjusted for relative humidity and PM_2·5_, respectively, and the secondary matched dataset matched on residential address at birth within 10 km and year of last menstrual period instead of last menstrual period plus or minus 7 days. Unit of exposure per 5°C increase in mean weekly ambient temperature. Horizontal bars represent 95% CIs. Significant associations between high ambient temperature and childhood acute lymphoblastic leukaemia are highlighted in black bold horizontal bars. OR=odds ratio. PM_2·5_=particulate matter less than 2·5 microns in aerodynamic diameter.

In our main analysis we observed a significant association between high ambient temperature in early pregnancy and an increased risk of childhood acute lymphoblastic leukaemia. Specifically, for each week from the 2 weeks preceding last menstrual period and through gestational week 20, there was a positive and significant association between ambient temperature and risk of acute lymphoblastic leukaemia (figure 1). The apex of the curve was in gestational week 8, where a 5°C increase in mean weekly temperature was associated with an OR for acute lymphoblastic leukaemia of 1·07 (95% CI 1·04–1·11). The sensitivity analyses restricted to the period with geocoding from street addresses, and using weekly averages based on daily minimum and maximum ambient temperatures yielded similar findings (figure 2; appendix pp 12-14).

In gestational week 8, we observed a slightly larger effect of high ambient temperature among Latino compared with non-Latino White children (figure 2), but the difference was not significant (p=0·24). Although the critical window of exposure in the Latino group reflected that of the main analysis (appendix p 15), the non-Latino White group had a wider critical window including the pre-pregnancy period (appendix p 16), albeit with wide confidence intervals including the point estimates of the Latino subgroup analysis.

There was a comparable and pronounced association between high ambient temperature and increased risk of acute lymphoblastic leukaemia diagnosed at 0–4 and 5–9 years of age (figure 2; p value of difference between the strata was 0·87). However, we observed no association between ambient temperature and risk of acute lymphoblastic leukaemia diagnosed at the age of 10–14 years (p values of difference between the strata 0–4 years and 10–14 years, and between 5–9 years and 10–14 years, were 0·0090 and 0·016, respectively). The critical window for the 0–4 years of age group was similar to that of the main analysis (appendix p 17). However, for the age 5–9 years subgroup, the critical window was wider, spanning from 13 weeks pre-pregnancy through gestational week 21 (appendix p 18). There was no critical window of exposure identified among those diagnosed at age 10–14 years (appendix p 19).

The results of the main analysis did not meaningfully change by adding relative humidity to the model (appendix p 20). However, when adding weekly PM_2·5_ to the model, the observed association between high ambient temperature and risk of acute lymphoblastic leukaemia was even more pronounced in early pregnancy (2394 cases and 115 587 controls; appendix p 21). In gestational week 8, there was a tendency of a greater harmful effect of high ambient temperature in the presence of high levels of air pollution; however, due to small sample sizes, there was little power to detect differences between the strata (p values of difference between the first and second PM_2·5_ tertile were 0·26, between the first and third PM_2·5_ tertile 0·31, and between the second and third PM_2·5_ tertile were 0·039).

**Figure 3:**
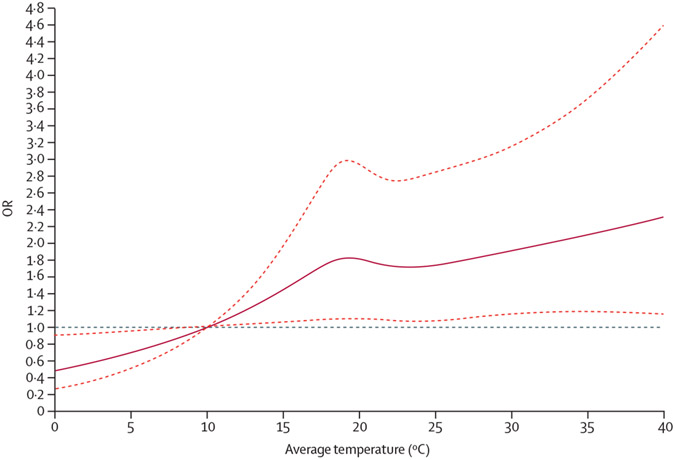
Non-linear analysis of ambient temperature and risk of childhood acute lymphoblastic leukaemia in gestational week 8 Results from cubic spline analysis with five knots. Mean weekly ambient temperature of 10°C used as reference. Accounted for race, ethnicity, birth order, maternal and paternal age, maternal education, Social Deprivation Index, date of last menstrual period plus or minus 7 days (ie, seasonality and time trend), and infant sex. The solid line represents the point estimates and the dotted lines represent the 95% CIs. OR=odds ratio.

The non-linear analysis of ambient temperature in gestational week 8 and risk of acute lymphoblastic leukaemia yielded a fairly linear association (figure 3). However, given the wide confidence intervals, the analysis did not rule out a progressively increased risk per unit increase of ambient temperature, nor an attenuation of the effect of ambient temperature at very high temperatures (p=0·14). Compared with a weekly mean temperature of 10°C, a mean temperature of 20°C was associated with an OR for acute lymphoblastic leukaemia of 1·80 (95% CI 1·10–2·94), a mean temperature of 30°C was associated with an OR of 1·90 (95% CI 1·16–3·14), and a mean temperature of 40°C was associated with an OR of 2·30 (95% CI 1·16–4·57).

Finally, we evaluated the critical windows of ambient temperature exposure using the secondary matched dataset controlling for residential address, while allowing for seasonal effects (appendix p 22). In total, 6188 cases and 24 434 controls were included in the secondary matched dataset. As with the main analysis, we observed a positive association between high ambient temperature in early pregnancy and risk of acute lymphoblastic leukaemia, with a peak in gestational week 0 (OR 1·03 [95% CI 1·01–1·04]). After adjusting for PM_2·5_, the peak association was shifted to gestational week 6 (OR 1·03 [95% CI 1·01–1·05]; appendix p 23).

## Discussion

In this first study to evaluate the potential association between ambient temperature during pregnancy and risk of cancer in infants, we observed a robust association between high ambient temperature in early pregnancy and risk of childhood acute lymphoblastic leukaemia in a set of models accounting for important confounders, humidity and air pollution, exposure contrast by time and space, and evaluation of a negative control exposure period. The strongest association between high ambient temperature and risk of acute lymphoblastic leukaemia was observed in gestational week 8.

Possible biological mechanisms underlying an association between high ambient temperature in early pregnancy and the risk of childhood leukaemia are unknown, but there are several plausible pathways as described in this Article. It is worth noting that the peak critical window of exposure was in gestational week 8, as hypothesised. The observed critical exposure window in early pregnancy will by our study design repeat itself every 12 months; at 4–8 months of age and 16–20 months of age, following this pattern. However, as there are no clear reasons why these narrow postnatal periods would be particularly vulnerable periods for lymphopoiesis and given that the majority of childhood acute lymphoblastic leukaemia cases have pre-leukaemic clones present at the time of birth,^8^ we believe that the most likely critical window is in the early pregnancy period.

Previous studies from California over the same study period as ours have observed both nitrogen oxides and PM_2·5_ during pregnancy to be associated with risk of childhood acute lymphoblastic leukaemia.^10,12^ Although it is challenging to prevent high ambient temperatures, air pollution is something that can be directly addressed. It was therefore of interest to assess whether the observed associations between heat and acute lymphoblastic leukaemia were attenuated after controlling for the potential mediating role of air pollution. The magnitude of the association remained largely the same. Notably, the positive association between exposure to high ambient temperature and risk of acute lymphoblastic leukaemia was more pronounced at higher air pollution levels. In other words, reducing air pollution might also help attenuate the association between heat exposure in pregnancy and childhood acute lymphoblastic leukaemia.

One of our sensitivity analyses evaluated a secondary matched dataset in which we effectively compared pregnancies at the same location and same year but at different stages of pregnancy in that year, as opposed to the main analyses that compared pregnancies at the same stage of pregnancy at the same time but at different geographical locations. By design, the secondary matched dataset was vulnerable to confounding due to seasonal patterns. The apex of the curve of the sensitivity analysis was shifted towards an earlier week compared with the main analysis. We interpret this shift as reflecting bias from seasonality, such as seasonal variation in air pollutants.^30^ We therefore conducted an analysis on the secondary matched dataset where we additionally adjusted for PM_2·5_. In this analysis, the apex of the curve was shifted to gestational week 6, closely resembling the pattern from the main analysis. Interpreted together with the main analysis, this sensitivity analysis strongly supports a positive association between ambient temperature in first trimester and risk of acute lymphoblastic leukaemia, and underscores that residual confounding due to risk factors related to residential address is unlikely to explain the results of the main analysis.

In the main analysis, we observed little effect of high ambient temperatures before pregnancy, other than what would be expected due to correlation of temperatures between weeks. The length of the lag-time between heat exposure and the initiation of leukaemogenesis is not known. Theoretically, prolonged exposure to heat stress might initiate maternal biological processes that will eventually affect lymphopoiesis, and there might be epigenetic modifications to sperm and ova relevant to leukemogenesis. This would affect the validity of interpreting the pre-pregnancy period as a negative control exposure period. However, it is reasonable to assume that the further away from the initiation of lymphopoiesis, the weaker the association between high ambient temperature and acute lymphoblastic leukaemia. Our findings from the main analysis of little to no association in the pre-pregnancy period offer support of a robust association between heat in the first trimester and risk of acute lymphoblastic leukaemia.

There was a more pronounced association between high ambient temperature and risk of acute lymphoblastic leukaemia among Latino than among non-Latino White subjects. If replicated, such differences might reflect disparate exposure to heat due to occupation and residence,^1-3^ or higher sensitivity to heat due to comorbidities. The critical window in the non-Latino White group showed a tendency of expanding longer into the pre-pregnancy period compared with the Latino group. The reason for this is unclear. Hypothetically, if non-Latino White women are less exposed to ambient heat than Latino women, a longer period of exposure might be necessary to trigger the biological processes associated with leukemogenesis. When stratified by age of onset, we observed a strong association between high ambient temperature in pregnancy and risk of early-onset childhood acute lymphoblastic leukaemia, but no association for late-onset childhood acute lymphoblastic leukaemia. This is in line with previous observations of early-onset childhood acute lymphoblastic leukaemia being more strongly linked with fetal insults than later onset childhood acute lymphoblastic leukaemia.^18^ The critical window for the subgroup diagnosed at age 5–9 years expanded into the pre-pregnancy period. We do not know why this pattern differed from those diagnosed at an earlier age. One theory is that the critical window here identified as pre-pregnancy might reflect early infant exposure (given the aforementioned 12-month cycle). Although the postnatal period is less relevant for the very early onset childhood acute lymphoblastic leukaemia cases, it might be a potential window of susceptibility for those diagnosed at early school ages. This would need to be investigated in future studies.

Our study has notable strengths. The population-based record-linkage between the California Cancer Registry and birth records allowed us to include close to all children with acute lymphoblastic leukaemia born in California, minimising selection bias. The controls were a random sample representative of the birth cohort in California each year. Some controls might in theory have moved out of California and since developed childhood acute lymphoblastic leukaemia before age 14 years, thus introducing control misclassification. However, childhood acute lymphoblastic leukaemia is rare, affecting 40 per million children,^5^ amounting to a maximum of a dozen misclassified controls, which is unlikely to affect our results. Recall bias is not possible because birth record data are collected before the diagnosis of acute lymphoblastic leukaemia. We used accurate ambient temperature data within a 1-km grid. In addition to robust adjustments for confounding, we carried out extensive sensitivity analyses to make sure that our estimates were not due to bias. Given that we identified a specific critical exposure window, it is unlikely that our findings are affected by time-invariant confounding factors.^28^ Finally, our sample size is large and unprecedented for an epidemiological study of childhood acute lymphoblastic leukaemia.

There are also inherent limitations that should be highlighted. When the study population is fixed by birth dates, fixed cohort bias might be introduced.^31^ However, we do not believe that this affected our results because we matched on last menstrual period. Another potential bias when evaluating offspring outcomes is live birth bias.^32^ High ambient temperature is associated with an increased risk of stillbirths,^4^ and if the fetuses lost were of greater risk of acute lymphoblastic leukaemia (eg, due to a common cause of fetal loss and acute lymphoblastic leukaemia such as smoking^33^) this would bias our results towards the null.^32^ The individual-level exposure to high ambient temperature might be modified by factors such as access to adequate air conditioning and line of work, but we did not have data to evaluate these factors. Also, geocoding was based on maternal residential address at the time of birth, and in situations in which participants had moved before giving birth there would be exposure misclassification (with the early pregnancy period more likely to be affected than the late pregnancy period). However, residential mobility has previously been shown to have little effect when evaluating critical exposure windows in pregnancy,^34^ and there is no reason to believe that this exposure misclassification would be differential by case or control status, therefore any bias would be towards the null.

Due to climate change, high ambient temperature is expected to be more common and intense over the coming decades, in the USA and worldwide.^1^ We have for the first time established a robust association between high ambient temperature during pregnancy and risk of childhood acute lymphoblastic leukaemia. Our study adds to a growing body of literature underscoring that high ambient temperature not only has immediate health effects, but might also be a cause of chronic diseases.

## Supplementary Material

1

## Figures and Tables

**Table:** Study population characteristics

	Entire study population (n=313 837)	cases (n=6258)	Controls (307 579)
Sex			
Female	139 144 (44·3%)	2773 (44·3%)	136 371 (44·3%)
Male	174 693 (55·7%)	3485 (55·7%)	171 208 (55·7%)
Race and ethnicity			
Latino	174 906 (55·7%)	3431 (54·8%)	171 475 (55·7%)
Non-Latino White	96 562 (30·8%)	1898 (30·3%)	94 664 (30·8%)
Non-Latino Black	10 121 (3·2%)	223 (3·6%)	9898 (3·2%)
Non-Latino Asian or Pacific Islander	30 959 (9·9%)	622 (9·9%)	30 337 (9·9%)
Other	1289 (0·4%)	84 (1·3%)	1205 (0·4%)
Year of birth			
1982–1995	135 097 (43·0%)	2661 (42·5%)	132,436 (43·1%)
1996–2005	119 012 (37·9%)	2371 (37·9%)	116,641 (37·9%)
2006–2015	59 728 (19·0%)	1226 (19·6%)	58,502 (19·0%)
Caesarean delivery	78 605 (25·0%)	1668 (26·7%)	76 937 (25·0%)
Singleton	305 954 (97·5%)	6097 (97·4%)	299 857 (97·5%)
Firstborn	123 695 (39·4%)	2485 (39·7%)	121 210 (39·4%)
Birthweight, g	3367·1 (569·3)	3413·1 (547·5)	3366–2 (569·7)
Gestational age at birth, days	277 (269–285)	277 (269–285)	277 (269–285)
Maternal age, years	27·4 (6·2)	27·9 (6·2)	27·4 (6·2)
Paternal age, years	30·2 (7·0)	30·6 (7·0)	30·2 (7·0)
Maternal education, years			
≤8	35 354 (11·3%)	587 (9·4%)	34 767 (11·3%)
9–11	48 597 (15·5%)	922 (14·7%)	47 675 (15·5%)
12	73 454 (23·4%)	1473 (23·5%)	71 981 (23·4%)
13–15	51 505 (16·4%)	1077 (17·2%)	50 428 (16·4%)
≥16	52 562 (16·7%)	1130 (18·1%)	51 432 (16·7%)
Census block group Social Deprivation			
First tertile (highest social deprivation)	104 297 (33·2%)	1998 (31·9%)	101 821 (33·1%)
Second tertile	104 702 (33·4%)	2113 (33·8%)	103 222 (33·6%)
Third tertile (lowest social deprivation)	104 426 (33·3%)	2146 (34·3%)	102 431 (33·3%)

Data are n (%), mean (SD), or median (IQR).

## Data Availability

Data analysed in the current study are not publicly available. Data dictionaries for both birth records and cancer registry data are available on the website of the California Department of Public Health We are unable to share the data due to restriction of our human subject protocol and data sharing policies set by the State of California. Researchers interested in obtaining the data can submit an application to the California Health and Human Services Agency Committee for the Protection of Human Subjects, as we did for this study. We welcome questions from other investigators or requests for additional analyses that are pertinent to the data presented in this Article. We are open to sharing the process of geocoding maternal residential addresses and linking geocodes to environmental exposures (eg, ambient temperature), as well as the codes for all statistical analyses, upon request to Prof Xiaomei Ma (xiaomei.ma@yale.edu) at any time.

## Research in context

### Evidence before this study

Due to climate change, periods of high ambient temperatures have become increasingly common worldwide and in the USA. Over the past decades, the incidence of childhood acute lymphoblastic leukaemia in the USA has steadily increased, especially among Latino children. The reason for the increased incidence is unclear. To evaluate existing literature on ambient temperature in pregnancy and risk of infant cancer, we searched PubMed for articles published in English (no restrictions in date published) using the terms “ambient temperature” AND “pregnancy” AND “cancer” (and relevant synonyms and medical subject heading terms). There were no studies on the subject. To gather evidence of tangential studies relevant for our study (eg, month of birth and risk of childhood leukaemia), we did scoping searches in PubMed, looked up reference lists of relevant original studies and review papers on childhood cancer aetiology, and used citation tools at Web of Science. Previous studies have observed that children with early-onset acute lymphoblastic leukaemia are more likely to be born during winter and spring, aligning with high ambient temperatures in the first trimester. Furthermore, several studies have linked inflammation during pregnancy (eg, due to infection or air pollution) to risk of childhood cancers. Finally, several studies have demonstrated that childhood acute lymphoblastic leukaemia has a prenatal origin, and it is suspected that the first trimester is a particularly critical exposure window as lymphopoiesis starts around gestational week 8.

### Added value of this study

Our study is the first to evaluate the association between ambient temperature during pregnancy and the risk of any subsequent infant cancer. We found that high ambient temperatures in early pregnancy—particularly around gestational week 8—were associated with increased risk of childhood acute lymphoblastic leukaemia. This finding was supported by our a-priori hypothesis that the strongest association would be observed around the time that lymphopoiesis starts, by sensitivity analyses in which we observed no effect of high ambient temperatures before pregnancy (a negative control exposure period), and in an alternatively matched dataset in which we assessed potential residual confounding due to residential address.

### Implications of all the available evidence

High ambient temperatures in early pregnancy might be associated with risk of childhood acute lymphoblastic leukaemia and might explain parts of previous observations of air pollution in early pregnancy and risk of childhood acute lymphoblastic leukaemia (high ambient temperatures as a common cause) and winter or spring births and risk of early-onset childhood acute lymphoblastic leukaemia (high ambient temperatures in the first trimester). These findings urgently call for more attention to how climate change, through increased frequency and intensity of days with high ambient temperatures, could negatively affect pregnancy and cause chronic diseases in offspring, particularly among racial and ethnic minority groups.
