# Supplementary material for: High ambient temperature in pregnancy and risk of childhood acute lymphoblastic leukaemia: an observational study
Source: Lancet Planet Health. Author manuscript; Available in PMC 2024 Jul 22. (PMC11260908; doi:10.1016/S2542-5196(24)00121-9)
Supplement: 1 [file NIHMS2007235-supplement-1.pdf]

THE LANCET
Planetary Health

Supplementary appendix

This appendix formed part of the original submission and has been peer reviewed. We post it as supplied by the authors.

Supplement to: Rogne T, Wang R, Wang P, et al. High ambient temperature in pregnancy and risk of childhood acute lymphoblastic leukaemia: an observational study. *Lancet Planet Health* 2024; **8:** e506–14.

SUPPLEMENTARY MATERIAL TO:

High Ambient Temperature in Pregnancy and Risk of Childhood Acute Lymphoblastic Leukemia

Tormod Rogne, MD PhD[1,2], Rong Wang, PhD[1], Pin Wang, PhD[3], Nicole C. Deziel, PhD[3], Catherine Metayer, MD, PhD[4], Joseph L. Wiemels, PhD[5], Kai Chen[3], Joshua L. Warren, PhD[6], PhD, Xiaomei Ma, MSc PhD[1]

Affiliations:
1) Department of Chronic Disease Epidemiology, Yale School of Public Health, New Haven, CT, USA
2) Center for Perinatal, Pediatric and Environmental Epidemiology, Yale School of Public Health, New Haven, CT, USA
3) Department of Environmental Health Sciences, Yale School of Public Health, New Haven, CT, USA
4) School of Public Health, University of California, Berkeley, CA, USA.
5) Center for Genetic Epidemiology, Department of Population and Public Health Sciences, Keck School of Medicine, University of Southern California, Los Angeles, CA, USA.
6) Department of Biostatistics, Yale School of Public Health, New Haven, CT, USA

SUPPLEMENTARY METHODS

Temperature Ascertainment

The Daymet method for calculating daily weather data in areas without weather stations relies on a combination of interpolation and extrapolation. It utilizes information from multiple instrumented locations, assigning weights to each site that consider the spatial and temporal relationships between the estimation area and the stations.[1] To improve robustness, based on pre-calculated arrays of station distances, the Daymet algorithm defines a search radius for every estimation location, sized to precisely capture the average number of input stations.[1] Strict cross-validation analysis was performed to objectively quantify the uncertainty, with a daily mean absolute error of 1.52 °C and 1.78 °C for daily minimum and maximum temperatures, respectively.[1]

Identification of Critical Windows of Exposure

The first stage association estimates were assumed to be normally distributed with mean equal to the true but unobserved association of interest, and variance equal to the squared standard error. The true associations were then modeled using the original CWVS methodology, which decomposes the single effect into continuous and binary components and uses a joint Gaussian process with temporal correlation structure to provide "smoothed" parameter estimation while conducting Bayesian variable selection output.

This second stage modeling resulted in estimates (i.e., posterior means), 95% credible intervals (i.e., equal tailed, quantile based), and relative importance estimates (i.e., marginal posterior inclusion probabilities) for each of the true associations of interest. CWVS was previously shown (Warren *et al.* 2020[2]) to provide improved estimation and statistical inference for identifying critical windows of susceptibility compared with 1, regression models that include all weekly effects at the same time with no regularization, 2, methods

which rely on Gaussian processes for temporal smoothing (similar to typical distributed lag methods), and 3, pure variable selection methods [i.e., stochastic search variable selection] which ignores the temporal correlation in the effects).[2–4] To account for correlation between exposure windows, CWVS combines variable selection with distributed lag modeling, and uses latent Gaussian processes with time series correlation structures to model the correlation between model parameters.

From this Bayesian analysis, we calculated posterior means as the point estimates and 95% quantile-based credible intervals to quantify uncertainty in the parameters (i.e., comparable to confidence intervals in the frequentist analysis setting). For consistency with the other analyses, the credible intervals are reported as confidence intervals.

To ensure that the new methodology performed as expected, we carried out an additional sensitivity analysis where in each weekly analysis we randomly shuffled the observed mean temperature values across the cases and controls (i.e., effectively breaking any association between temperature and ALL risk). Under this randomization of exposure, we expected to not observe any statistically significant association from the second stage meta-regression (i.e., credible intervals that excluded one). This is also what we observed.

Critical Window Variable Selection (CWVS) Meta-Regression

$$\hat{\alpha}_t|\alpha_t \sim \mathrm{N}(\alpha_t, \hat{\sigma}_t^2), t = 1, \dots, n$$

- $\hat{\alpha}_t$: Estimated effect for week *t* from the first stage regression analyses
- $\hat{\sigma}_t^2$: Variance of estimate from the first stage regression analyses (squared standard error)
- n: Total number of weeks in the first stage regression analyses
- α_t: True but unobserved effect of interest defined as $\alpha_t = \theta_t \gamma_t$
    - θ_t: Continuous component defined as $\theta_t = A_{11}\delta_{1t}$

- γ_t: Binary component defined as $\gamma_t|\pi_t \sim \text{Bernoulli}(\pi_t)$ where $\Phi^{-1}(\pi_t) = A_{21}\delta_{1t} + A_{22}\delta_{2t}$

- δ_{jt}: Parameters that account for temporal correlation and correlation between the continuous and binary components; modeled using autoregressive prior distributions
    - $\delta_{j1}|\rho_j \sim \text{N}\left(0, 1 - \rho_j^2\right)$, $\delta_{jt}|\rho_j, \delta_{j,t-1} \sim \text{N}\left(\rho_j \delta_{j,t-1}, 1\right), t = 2, \dots, n; j = 1,2$
- Hyperprior distributions:
    - ρ_j: Temporal correlation parameters; $\rho_j \sim \text{Uniform}(0,1), j = 1,2$
    - A_{jj}: Variance parameters; $A_{jj} \sim \text{Uniform}(0,100), j = 1,2$
    - A_{21}: Cross-covariance parameter; $A_{21} \sim \text{N}(0,100^2)$
- The CWVS methodology was originally introduced in Warren et al. (2020).[2]

SUPPLEMENTARY TABLES

Supplementary Table 1. Number of subjects included in the main analysis, by week.

	Cases	Controls
Before Pregnancy Week 13	2764	132923
Before Pregnancy Week 12	2772	132528
Before Pregnancy Week 11	2818	134145
Before Pregnancy Week 10	2752	131777
Before Pregnancy Week 9	2742	131679
Before Pregnancy Week 8	2736	130984
Before Pregnancy Week 7	2766	131969
Before Pregnancy Week 6	2697	129260
Before Pregnancy Week 5	2698	129516
Before Pregnancy Week 4	2683	129003
Before Pregnancy Week 3	2681	127629
Before Pregnancy Week 2	2686	128145
Before Pregnancy Week 1	2676	128366
Pregnancy Week 0	2687	129056
Pregnancy Week 1	2675	128540
Pregnancy Week 2	2740	131062
Pregnancy Week 3	2691	129304
Pregnancy Week 4	2682	129057
Pregnancy Week 5	2676	128348
Pregnancy Week 6	2724	130154
Pregnancy Week 7	2643	126902
Pregnancy Week 8	2672	127988
Pregnancy Week 9	2647	127358
Pregnancy Week 10	2704	129798
Pregnancy Week 11	2708	130119
Pregnancy Week 12	2669	128943
Pregnancy Week 13	2656	128508
Pregnancy Week 14	2656	127425
Pregnancy Week 15	2681	129206
Pregnancy Week 16	2645	127674
Pregnancy Week 17	2650	128118
Pregnancy Week 18	2682	129475
Pregnancy Week 19	2758	132473
Pregnancy Week 20	2676	128936
Pregnancy Week 21	2710	130542
Pregnancy Week 22	2715	130409
Pregnancy Week 23	2775	133252
Pregnancy Week 24	2796	133660
Pregnancy Week 25	2742	131655
Pregnancy Week 26	2744	131899
Pregnancy Week 27	2751	131620
Pregnancy Week 28	2793	133801
Pregnancy Week 29	2761	132316
Pregnancy Week 30	2758	132047
Pregnancy Week 31	2732	130414
Pregnancy Week 32	2798	132116
Pregnancy Week 33	2705	127465
Pregnancy Week 34	2685	125559
Pregnancy Week 35	2674	123537
Pregnancy Week 36	2608	117183
Pregnancy Week 37	2491	107296
Pregnancy Week 38	2252	89114
Pregnancy Week 39	1774	56967
Pregnancy Week 40	1113	23154
Pregnancy Week 41	572	5974
Pregnancy Week 42	234	1055

SUPPLEMENTARY FIGURES

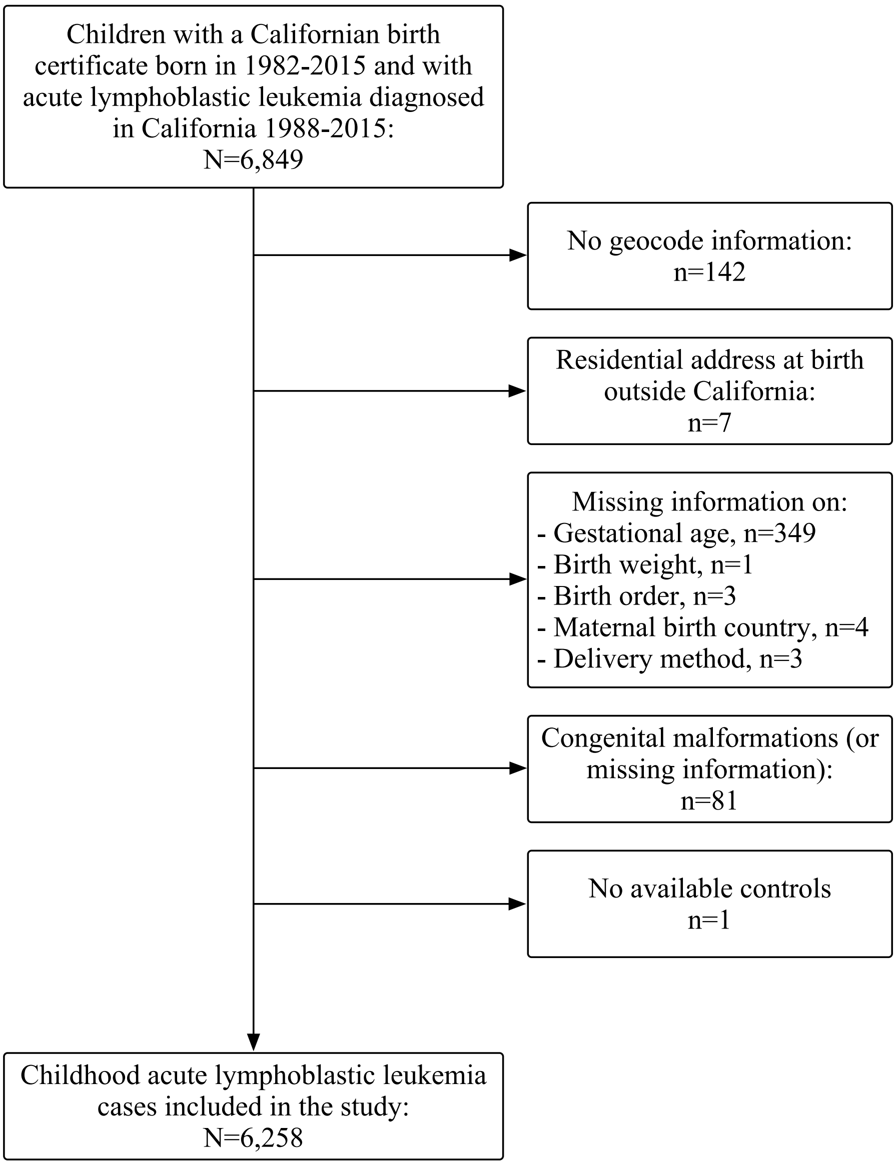

Supplementary Figure 1. Flowchart of selection of cases.

Legend: The figure illustrates the number of eligible cases that were eventually included in our study. For the majority of the analyses, each case was matched to up to 50 controls (matching on sex, race, ethnicity, and date of mother's last menstrual period ±7 days), totaling 307,579 controls. In the secondary matched dataset (see main text), each case was matched to up to 4 controls (matching on sex, race, ethnicity, year of mother's last menstrual period, and residential address at birth within 10 km); here, 71 cases did not have available controls, for a final total of 6,188 cases and 24,434 controls.

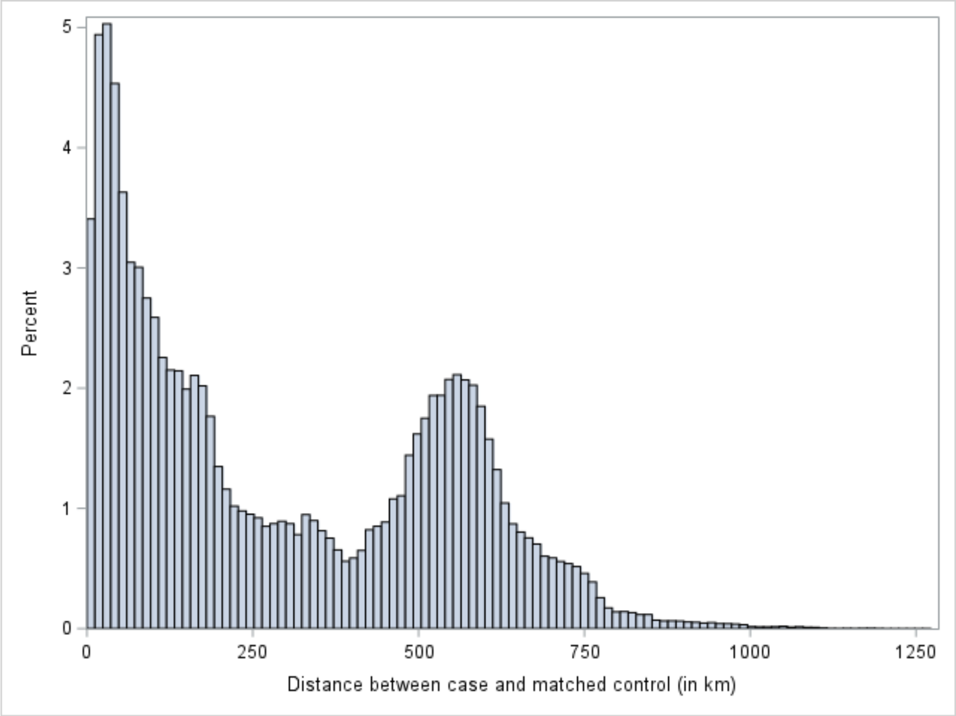

Supplementary Figure 2. Distance between cases and matched controls.

Legend: Presentation of the distribution of the distance between cases and matched controls in the main matched dataset. The minimum and maximum distance was 0 km and 1,269 km, respectively, with a median of 217 km.

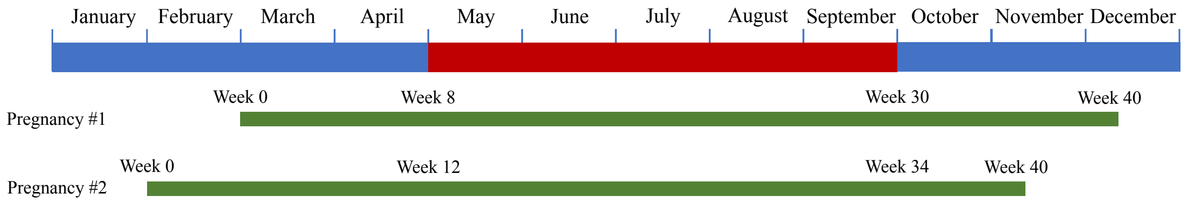

Supplementary Figure 3. Illustration of weeks of pregnancy included in analysis within the warm period.

Legend: For all analyses except the sensitivity analysis that uses the secondary matched dataset, the analyses are restricted to the warm period (highlighted in red). Thus, for a given gestational week under analysis, this week has to be within the warm period. In Pregnancy #1, gestational weeks 8 through 30 will be included in the analyses, while for Pregnancy #2, gestational weeks 12 through 34 will be considered.

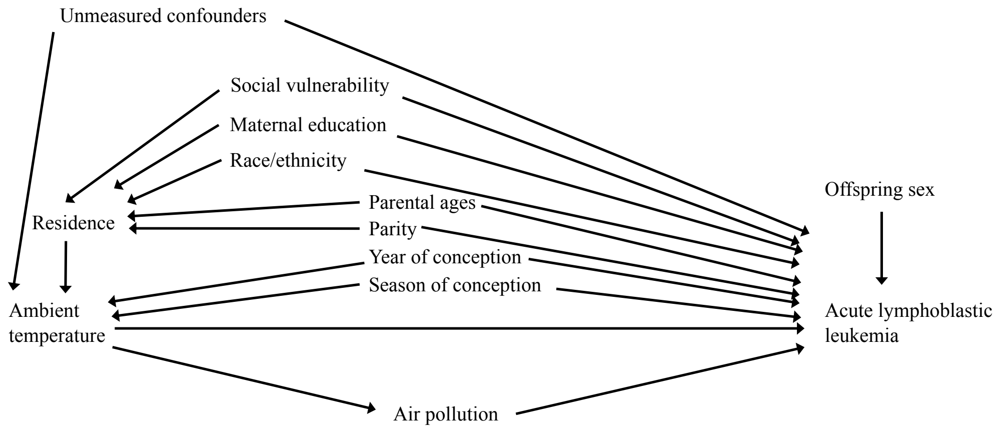

Supplementary Figure 4. Directed acyclic graph of ambient temperature exposure during pregnancy and risk of childhood acute lymphoblastic leukemia.

Legend: This simplified directed acyclic graph represents the hypothesized relationship between the relevant covariates considered in this study and risk of childhood acute lymphoblastic leukemia. Note that Social Vulnerability Index is at the population-level and captures educational level and percent racial/ethnic minority, among other things (see main text for details); for simplicity, arrows between social vulnerability and other covariates than residence and the outcome have not been drawn. Similarly, while all arrows directed to ambient temperature also affect air pollution, these latter arrows have not been drawn.

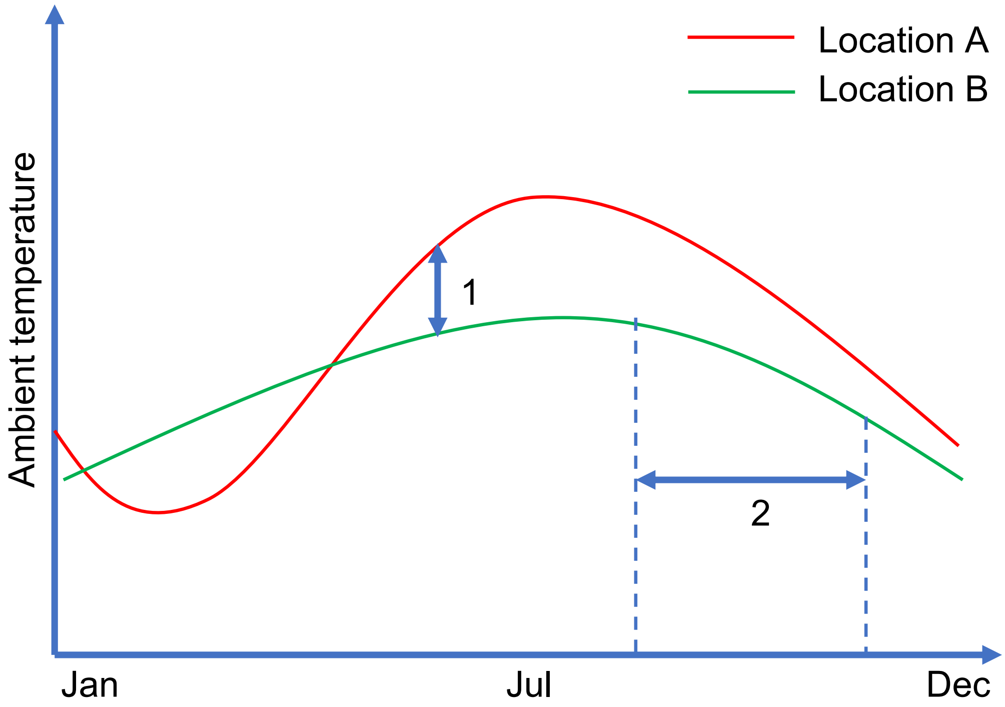

Supplementary Figure 5. Comparison of main and secondary matching strategies.

Legend: In the main matching strategy, **1**, we compared pregnancies occurring at the same time but in different locations, while in the secondary matching strategy, **2**, we compared pregnancies occurring in the same location and in the same year, but at different times during that year.

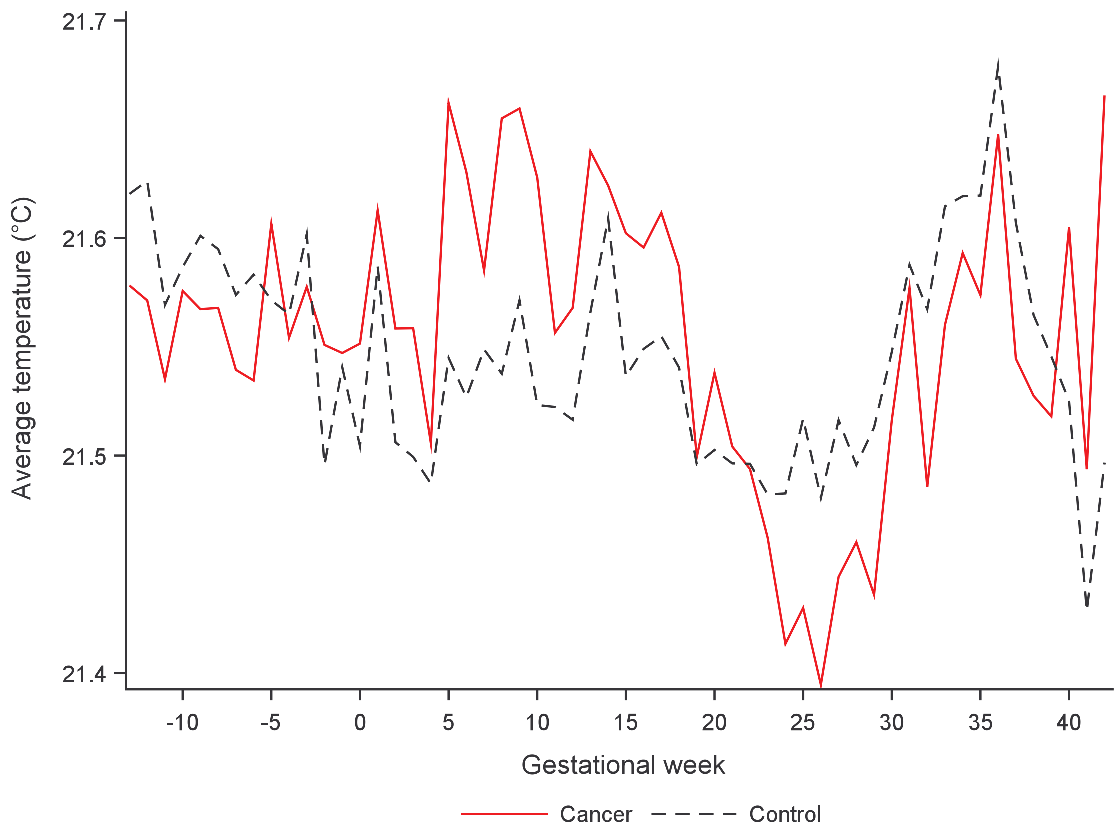

Supplementary Figure 6. Mean weekly temperature by cases and controls.

Legend: Weekly means based on daily means in the warm season of cancer cases and controls matched on sex, race or ethnicity, and date of last menstrual period.

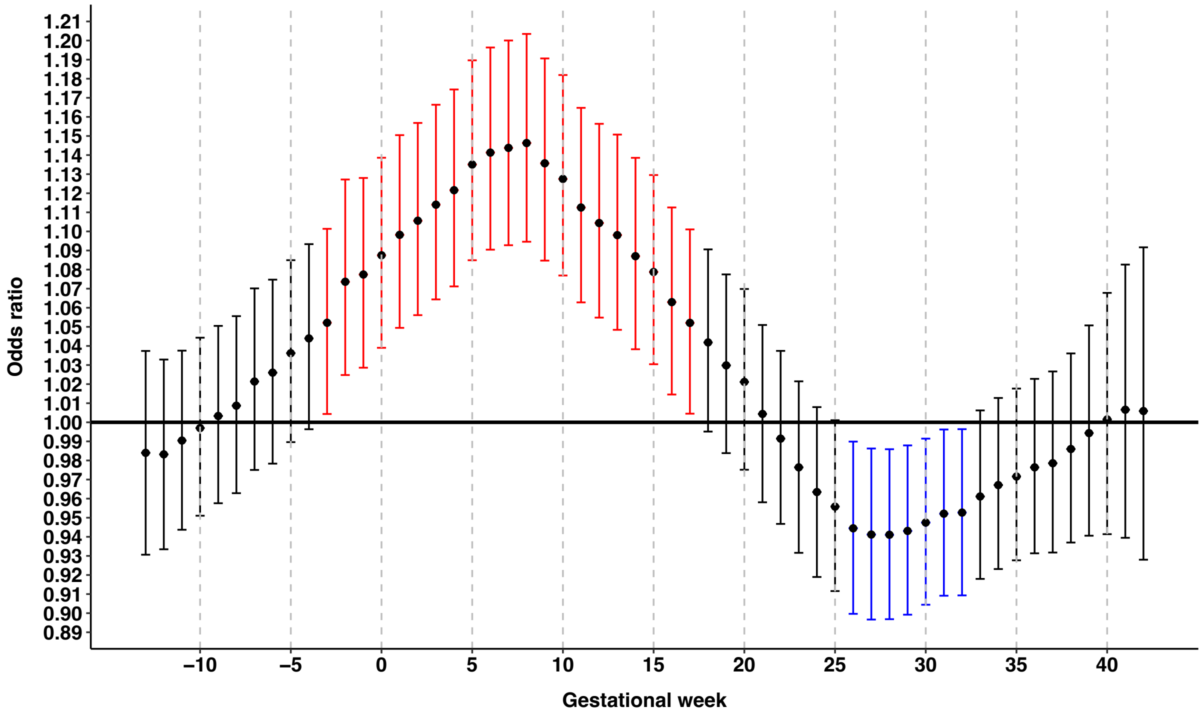

Supplementary Figure 7. Ambient temperature and risk of childhood acute lymphoblastic leukemia among subjects with geocoding based on street address.

Legend: Sensitivity analysis of the main analysis (Figure 1 in the main text) where this analysis is restricted to subjects where street addresses were used for geocoding (1997 and onwards), as opposed to zip codes. Results from the two-stage Bayesian meta-regression analysis of ambient temperature and risk of childhood acute lymphoblastic leukemia. Accounted for race, ethnicity, birth order, maternal and paternal age, maternal education, Social Vulnerability Index, date of LMP ±7 days (i.e., seasonality and time trend), and offspring sex. Unit of exposure per 5 °C increase in mean weekly ambient temperature. Vertical bars represent 95% confidence intervals. Statistically significant positive and negative associations between ambient temperature and childhood acute lymphoblastic leukemia are highlighted in red and blue, respectively.

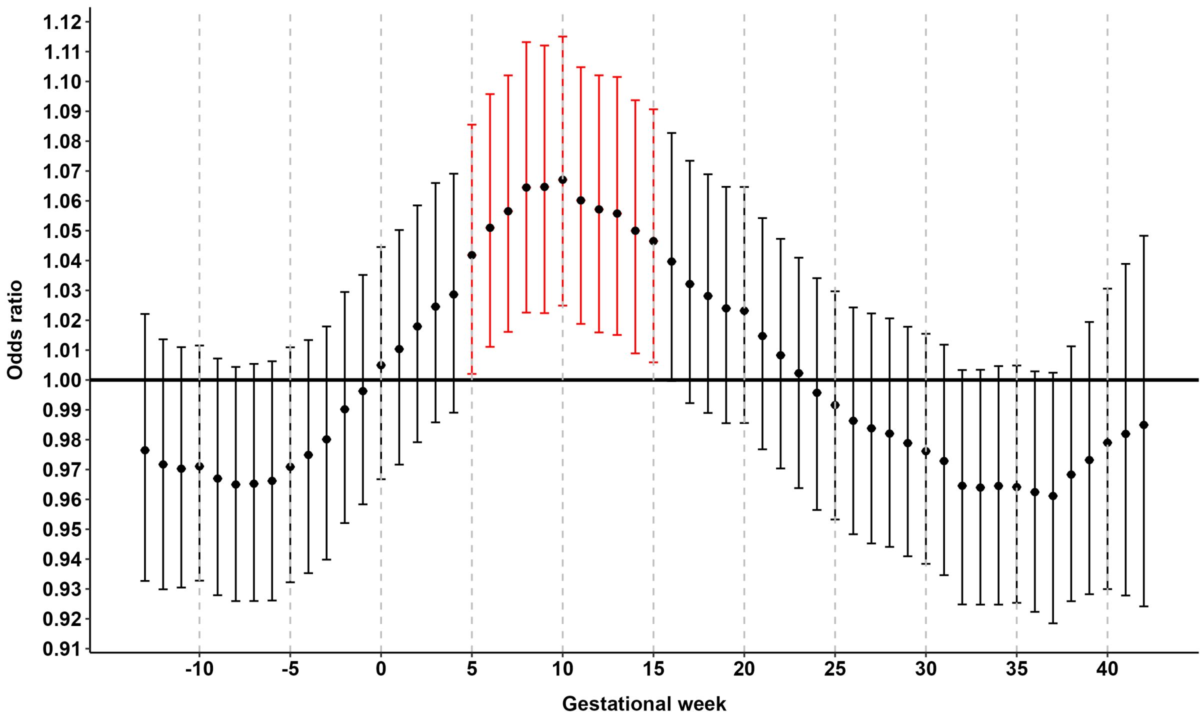

Supplementary Figure 8. Ambient temperature based on daily minimum temperatures and risk of childhood acute lymphoblastic leukemia.

Legend: Sensitivity analysis of the main analysis (Figure 1 in the main text) where this analysis uses weekly averaged temperatures that are based on daily minimum temperatures. Results from the two-stage Bayesian meta-regression analysis of ambient temperature and risk of childhood acute lymphoblastic leukemia. Accounted for race, ethnicity, birth order, maternal and paternal age, maternal education, Social Vulnerability Index, date of LMP ±7 days (i.e., seasonality and time trend), and offspring sex. Unit of exposure per 5 °C increase in mean weekly ambient temperature. Vertical bars represent 95% confidence intervals. Statistically significant associations between high ambient temperature and childhood acute lymphoblastic leukemia highlighted in red.

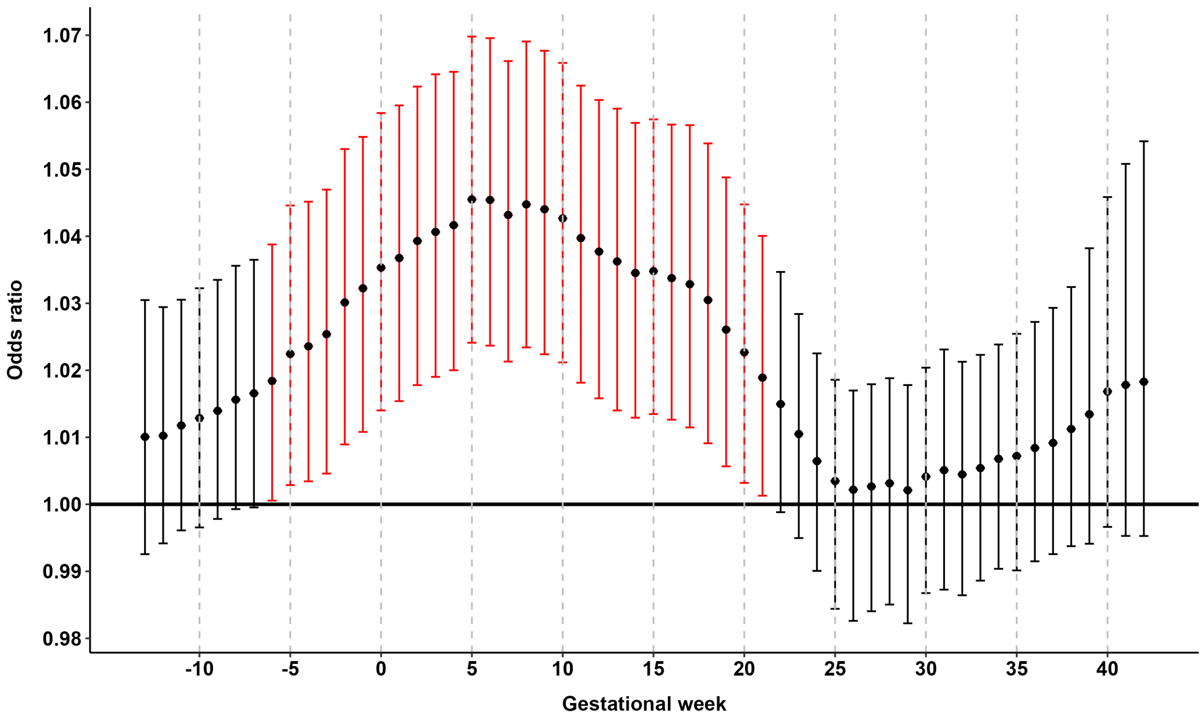

Supplementary Figure 9. Ambient temperature based on daily maximum temperatures and risk of childhood acute lymphoblastic leukemia.

Legend: Sensitivity analysis of the main analysis (Figure 1 in the main text) where this analysis uses weekly averaged temperatures that are based on daily maximum temperatures. Results from the two-stage Bayesian meta-regression analysis of ambient temperature and risk of childhood acute lymphoblastic leukemia. Accounted for race, ethnicity, birth order, maternal and paternal age, maternal education, Social Vulnerability Index, date of LMP ±7 days (i.e., seasonality and time trend), and offspring sex. Unit of exposure per 5 °C increase in mean weekly ambient temperature. Vertical bars represent 95% confidence intervals. Statistically significant associations between high ambient temperature and childhood acute lymphoblastic leukemia highlighted in red.

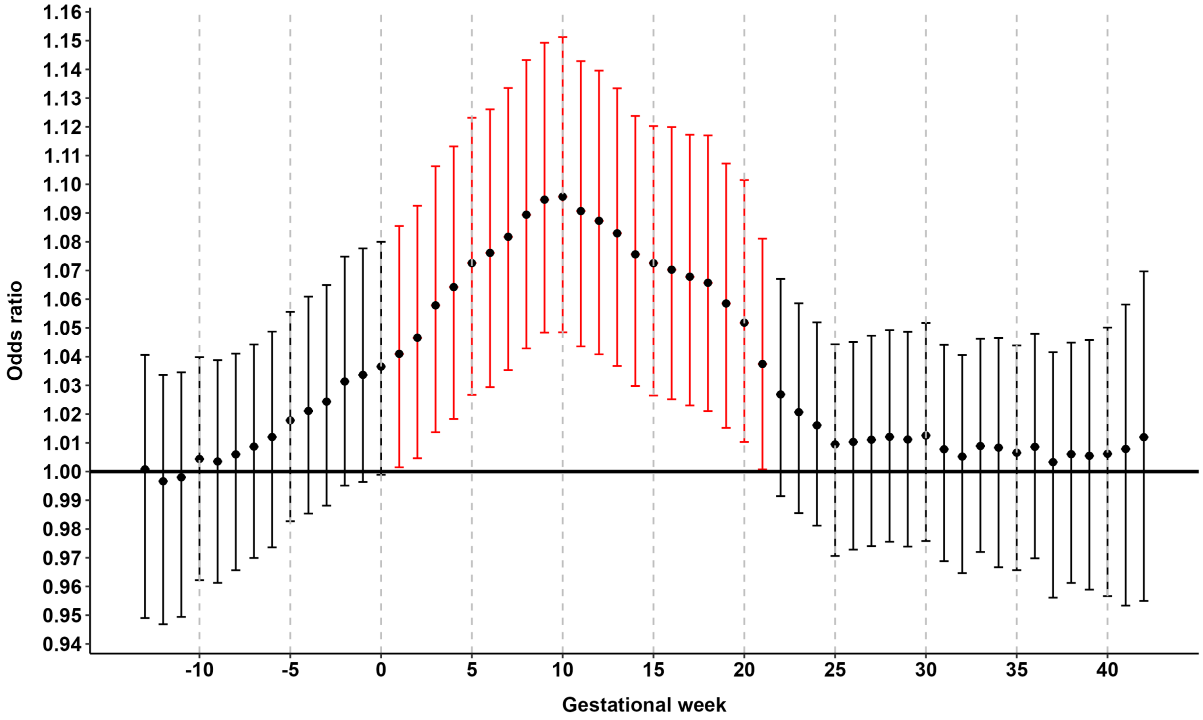

Supplementary Figure 10. Ambient temperature and risk of childhood acute lymphoblastic leukemia in the Latino subgroup.

Legend: Subgroup analysis of the main analysis (Figure 1 in the main text) where this analysis is restricted to the Latino subgroup. Results from the two-stage Bayesian meta-regression analysis of ambient temperature and risk of childhood acute lymphoblastic leukemia. Accounted for race, ethnicity, birth order, maternal and paternal age, maternal education, Social Vulnerability Index, date of LMP ±7 days (i.e., seasonality and time trend), and offspring sex. Unit of exposure per 5 °C increase in mean weekly ambient temperature. Vertical bars represent 95% confidence intervals. Statistically significant associations between high ambient temperature and childhood acute lymphoblastic leukemia highlighted in red.

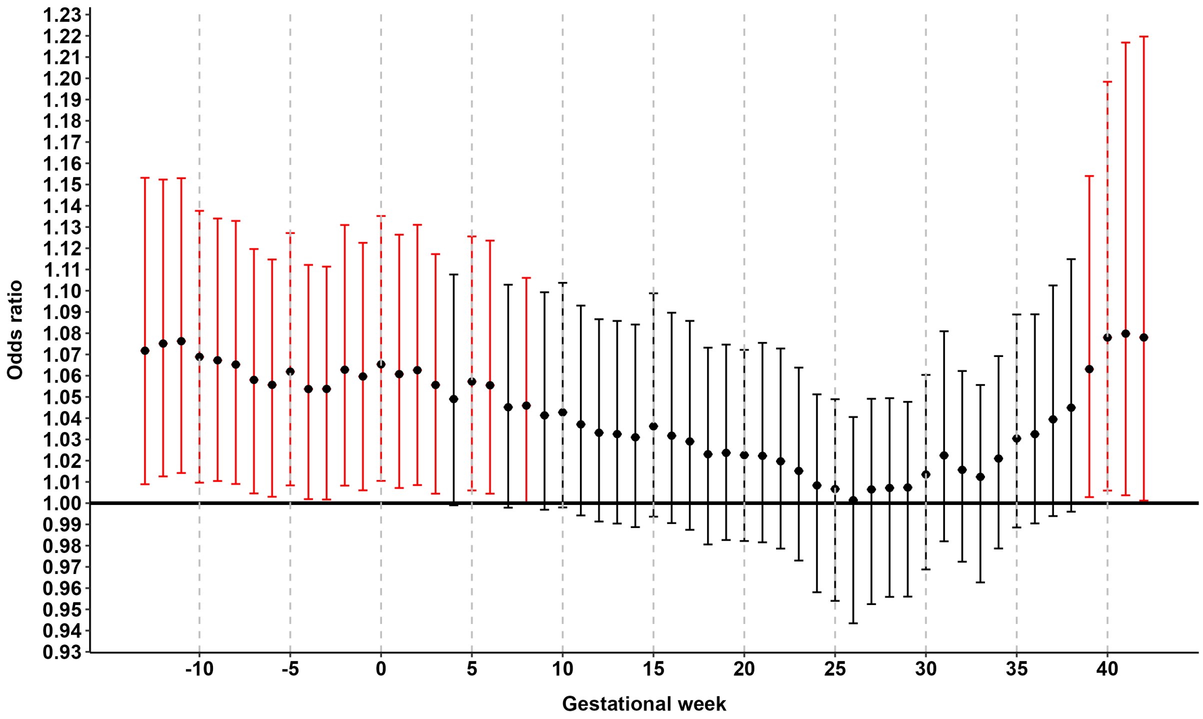

Supplementary Figure 11. Ambient temperature and risk of childhood acute lymphoblastic leukemia in the non-Latino White subgroup.

Legend: Subgroup analysis of the main analysis (Figure 1 in the main text) where this analysis is restricted to the non-Latino White subgroup. Results from the two-stage Bayesian meta-regression analysis of ambient temperature and risk of childhood acute lymphoblastic leukemia. Accounted for race, ethnicity, birth order, maternal and paternal age, maternal education, Social Vulnerability Index, date of LMP ±7 days (i.e., seasonality and time trend), and offspring sex. Unit of exposure per 5 °C increase in mean weekly ambient temperature. Vertical bars represent 95% confidence intervals. Statistically significant associations between high ambient temperature and childhood acute lymphoblastic leukemia highlighted in red.

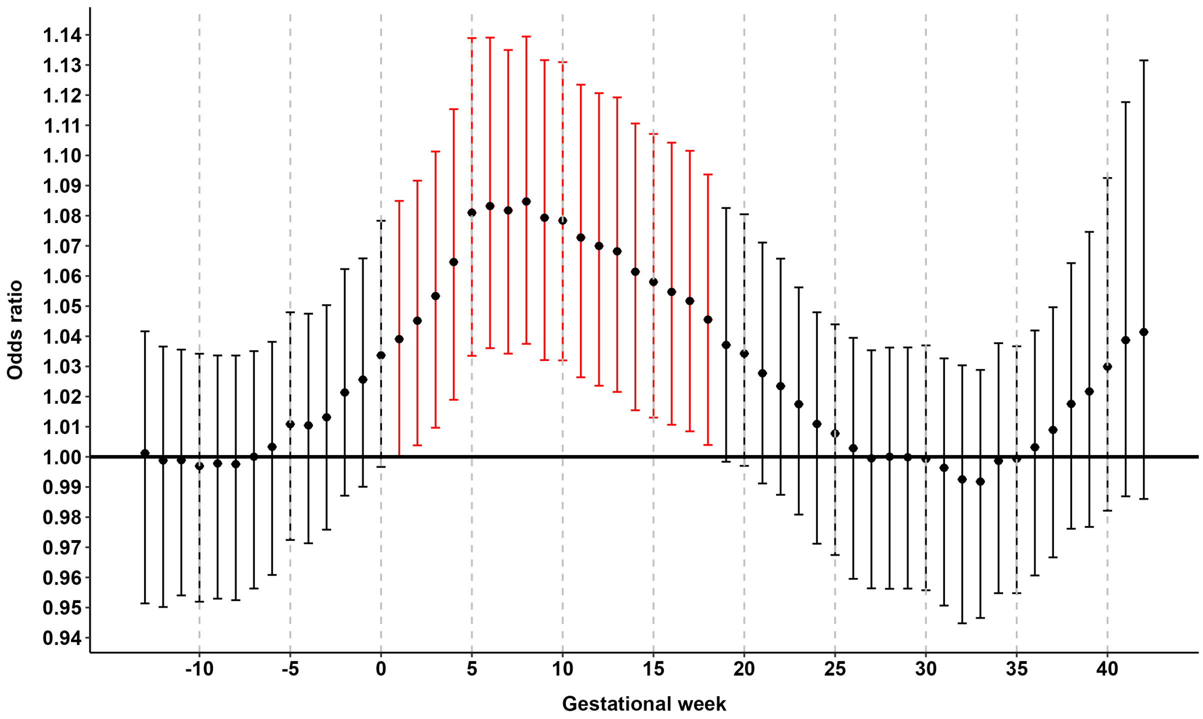

Supplementary Figure 12. Ambient temperature and risk of childhood acute lymphoblastic leukemia among those diagnosed between 0 and 4 years of age.

Legend: Subgroup analysis of the main analysis (Figure 1 in the main text) where this analysis is restricted to those diagnosed between 0 and 4 years of age. Results from the two-stage Bayesian meta-regression analysis of ambient temperature and risk of childhood acute lymphoblastic leukemia. Accounted for race, ethnicity, birth order, maternal and paternal age, maternal education, Social Vulnerability Index, date of LMP ±7 days (i.e., seasonality and time trend), and offspring sex. Unit of exposure per 5 °C increase in mean weekly ambient temperature. Vertical bars represent 95% confidence intervals. Statistically significant associations between high ambient temperature and childhood acute lymphoblastic leukemia highlighted in red.

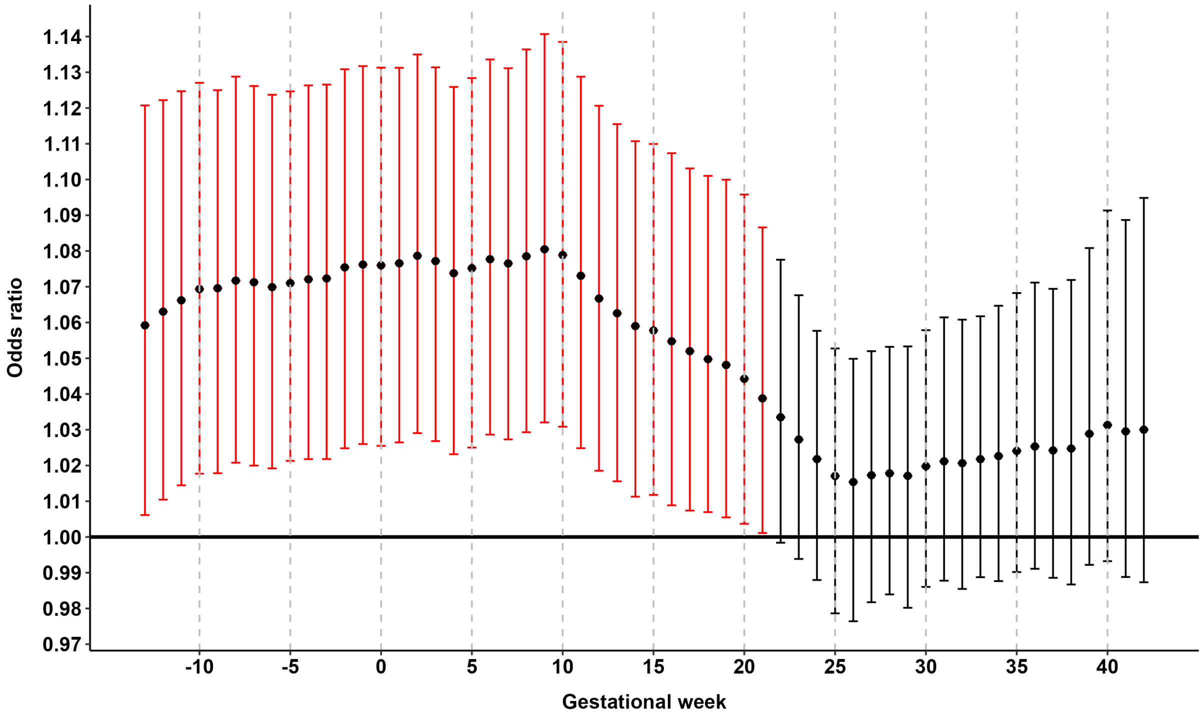

Supplementary Figure 13. Ambient temperature and risk of childhood acute lymphoblastic leukemia among those diagnosed between 5 and 9 years of age.

Legend: Subgroup analysis of the main analysis (Figure 1 in the main text) where this analysis is restricted to those diagnosed between 5 and 9 years of age. Results from the two-stage Bayesian meta-regression analysis of ambient temperature and risk of childhood acute lymphoblastic leukemia. Accounted for race, ethnicity, birth order, maternal and paternal age, maternal education, Social Vulnerability Index, date of LMP ±7 days (i.e., seasonality and time trend), and offspring sex. Unit of exposure per 5 °C increase in mean weekly ambient temperature. Vertical bars represent 95% confidence intervals. Statistically significant associations between high ambient temperature and childhood acute lymphoblastic leukemia highlighted in red.

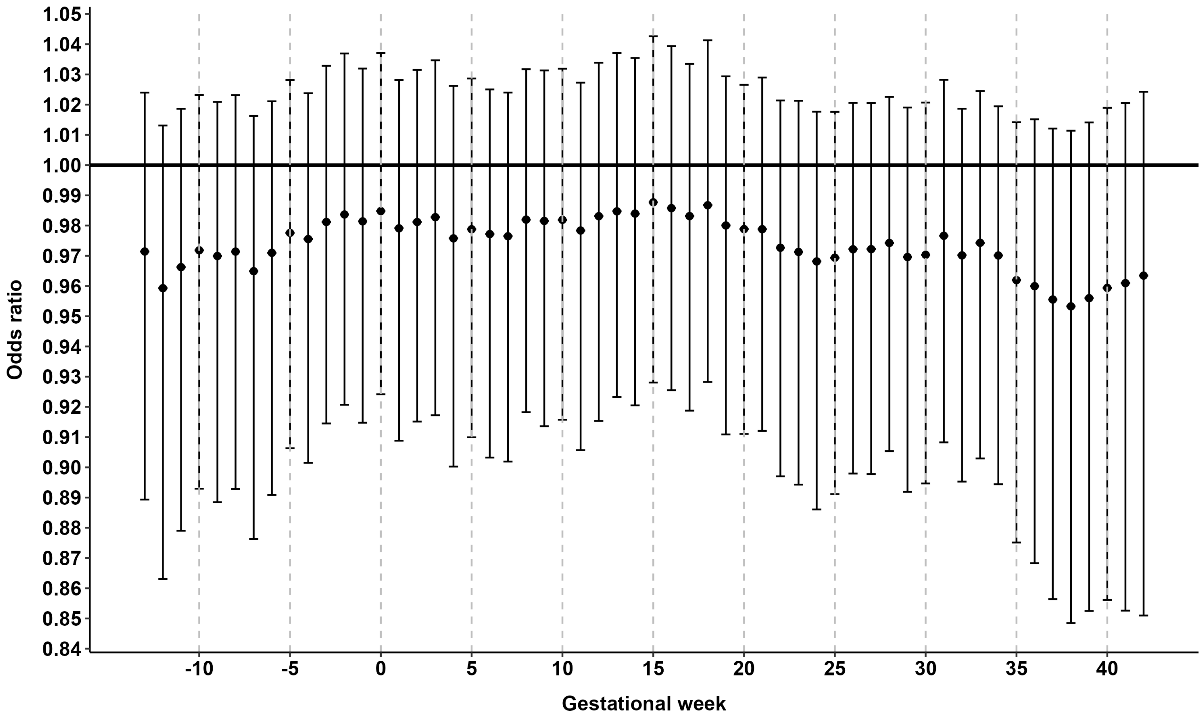

Supplementary Figure 14. Ambient temperature and risk of childhood acute lymphoblastic leukemia among those diagnosed between 10 and 14 years of age.

Legend: Subgroup analysis of the main analysis (Figure 1 in the main text) where this analysis is restricted to those diagnosed between 10 and 14 years of age. Results from the two-stage Bayesian meta-regression analysis of ambient temperature and risk of childhood acute lymphoblastic leukemia. Accounted for race, ethnicity, birth order, maternal and paternal age, maternal education, Social Vulnerability Index, date of LMP ±7 days (i.e., seasonality and time trend), and offspring sex. Unit of exposure per 5 °C increase in mean weekly ambient temperature. Vertical bars represent 95% confidence intervals. Statistically significant associations between high ambient temperature and childhood acute lymphoblastic leukemia highlighted in red.

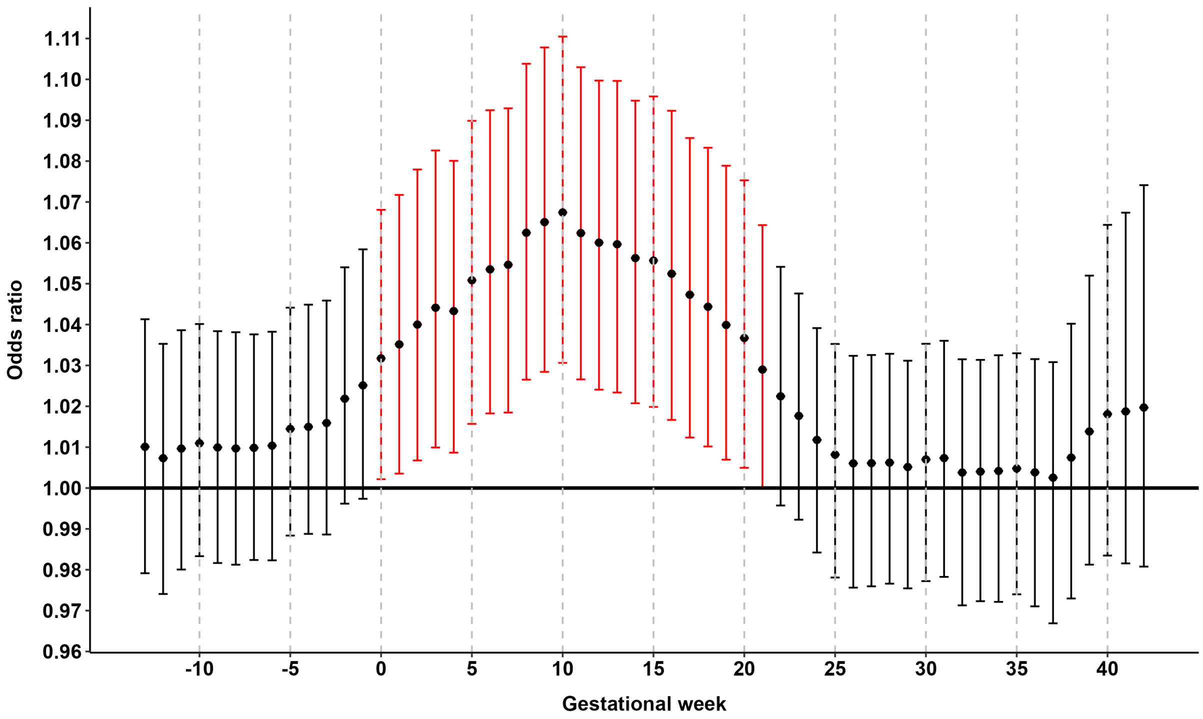

Supplementary Figure 15. Ambient temperature and risk of childhood acute lymphoblastic leukemia while adjusting for relative humidity.

Legend: Sensitivity analysis of the main analysis (Figure 1 in the main text) where this analysis additionally adjusts for relative humidity. Results from the two-stage Bayesian meta-regression analysis of ambient temperature and risk of childhood acute lymphoblastic leukemia. Accounted for race, ethnicity, birth order, maternal and paternal age, maternal education, Social Vulnerability Index, date of LMP ±7 days (i.e., seasonality and time trend), offspring sex, and relative humidity. Unit of exposure per 5 °C increase in mean weekly ambient temperature. Vertical bars represent 95% confidence intervals. Statistically significant associations between high ambient temperature and childhood acute lymphoblastic leukemia highlighted in red.

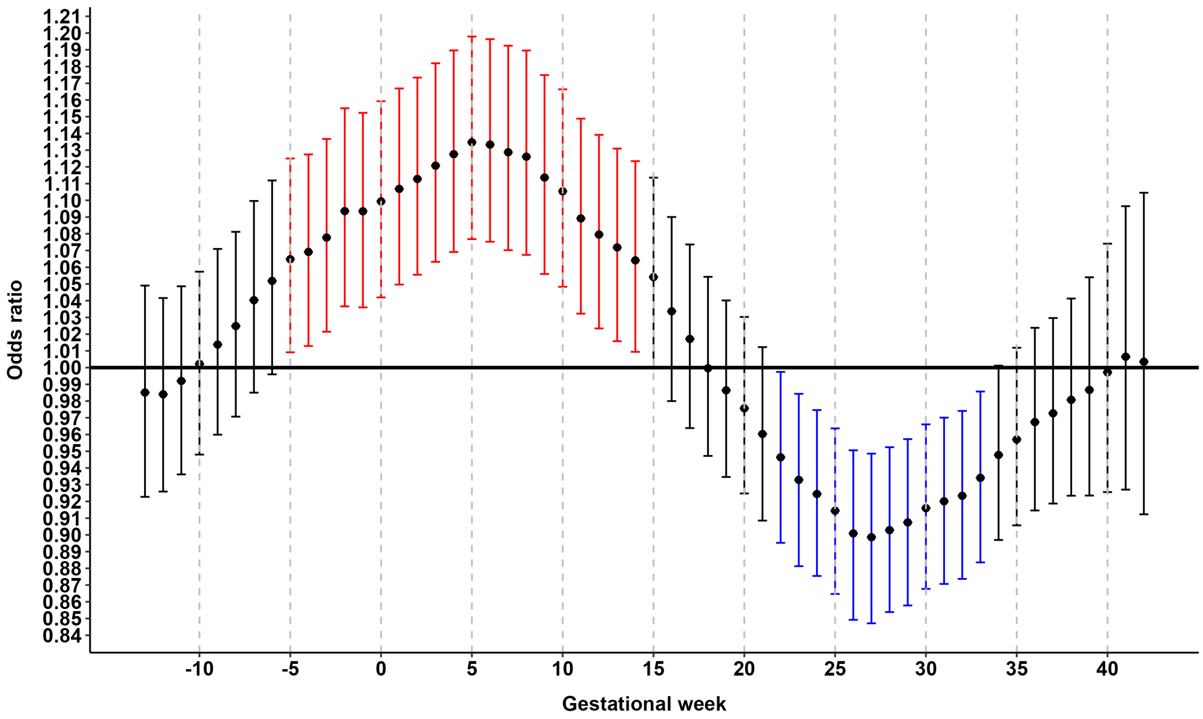

Supplementary Figure 16. Ambient temperature and risk of childhood acute lymphoblastic leukemia while adjusting for $PM_{2.5}$.

Legend: Sensitivity analysis of the main analysis (Figure 1 in the main text) where this analysis additionally adjusts for $PM_{2.5}$. Results from the two-stage Bayesian meta-regression analysis of ambient temperature and risk of childhood acute lymphoblastic leukemia. Accounted for race, ethnicity, birth order, maternal and paternal age, maternal education, Social Vulnerability Index, date of LMP ±7 days (i.e., seasonality and time trend), offspring sex, and $PM_{2.5}$. Unit of exposure per 5 °C increase in mean weekly ambient temperature. Vertical bars represent 95% confidence intervals. Statistically significant positive and negative associations between ambient temperature and childhood acute lymphoblastic leukemia are highlighted in red and blue, respectively. $PM_{2.5}$, particulate matter less than 2.5 microns in aerodynamic diameter.

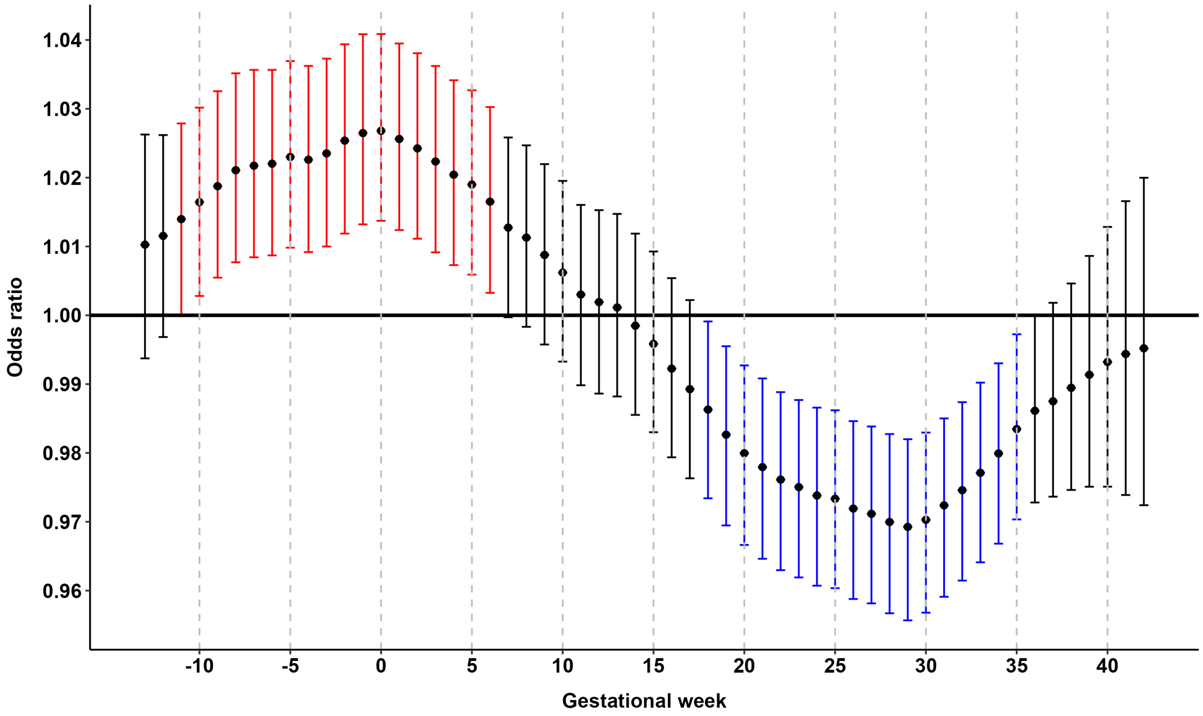

Supplementary Figure 17. Ambient temperature and risk of childhood acute lymphoblastic leukemia using the alternative dataset matched on residential address at birth.

Legend: Results from the sensitivity analysis using the secondary matched dataset where cases and controls were matched based on *residential address at birth within 10 km*, sex, race, ethnicity, and *year of last menstrual period* (instead of week). This analysis compared pregnancies at the same location, but at different stages of pregnancy within the same year, as compared with the main analysis which additionally accounted for week of last menstrual period but not for geographic location. The two-stage Bayesian meta-regression analysis of ambient temperature and risk of childhood acute lymphoblastic leukemia was applied. Accounted for race, ethnicity, birth order, maternal and paternal age, maternal education, neighborhood poverty, date of LMP +/- 365 days (i.e., seasonality and time trend), offspring sex, and place of residence within 10 km. Unit of exposure per 5 °C increase in mean weekly ambient temperature. Vertical bars represent 95% confidence intervals. Statistically significant positive and negative associations between ambient temperature and childhood acute lymphoblastic leukemia are highlighted in red and blue, respectively.

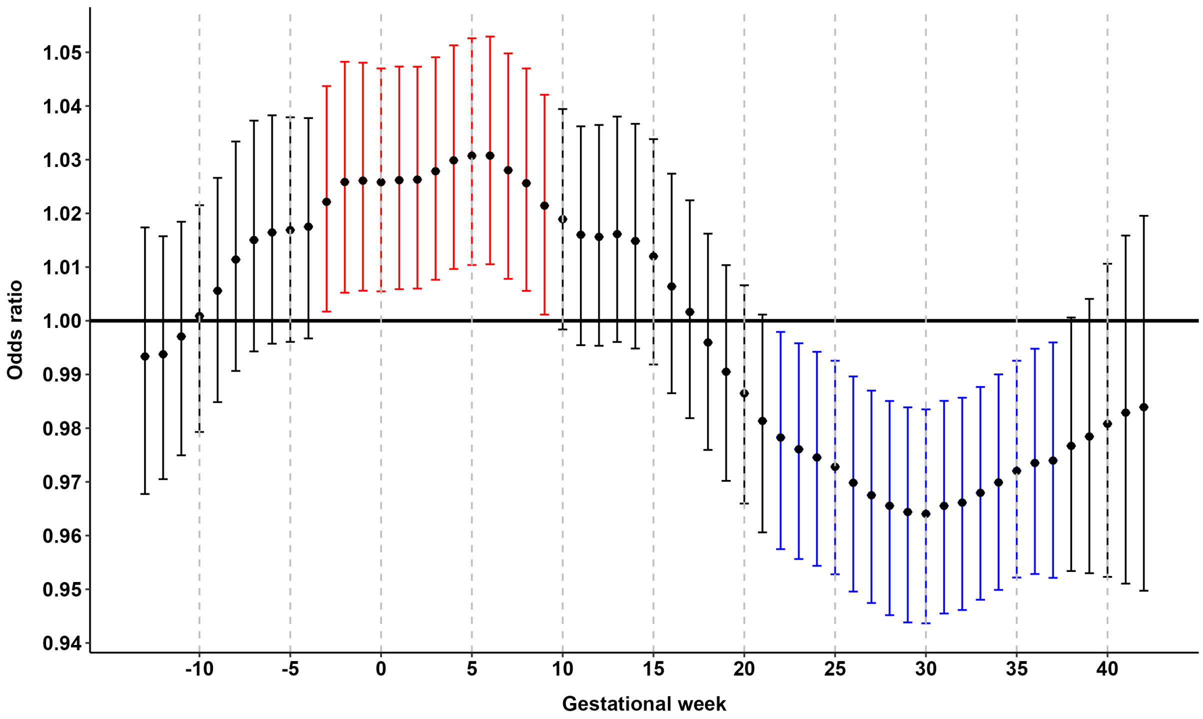

Supplementary Figure 18. Ambient temperature and risk of childhood acute lymphoblastic leukemia using the alternative dataset matched on residential address at birth while adjusting for $PM_{2.5}$.

Legend: Results from the sensitivity analysis using the secondary matched dataset where cases and controls were matched based on *residential address at birth within 10 km*, sex, race, ethnicity, and *year of last menstrual period* (instead of week), and additionally adjusting for $PM_{2.5}$. This analysis compared pregnancies at the same location, but at different stages of pregnancy within the same year, as compared with the main analysis which additionally accounted for week of last menstrual period but not for geographic location. The two-stage Bayesian meta-regression analysis of ambient temperature and risk of childhood acute lymphoblastic leukemia was applied. Accounted for race, ethnicity, birth order, maternal and paternal age, maternal education, neighborhood poverty, date of LMP +/- 365 days (i.e., seasonality and time trend), offspring sex, place of residence within 10 km, and $PM_{2.5}$. Unit of exposure per 5 °C increase in mean weekly ambient temperature. Vertical bars represent 95% confidence intervals. Statistically significant positive and negative associations between ambient temperature and childhood acute lymphoblastic leukemia are highlighted in red and blue, respectively. $PM_{2.5}$, particulate matter less than 2.5 microns in aerodynamic diameter.

REFERENCES

1 Thornton PE, Shrestha R, Thornton M, Kao SC, Wei Y, Wilson BE. Gridded daily weather data for North America with comprehensive uncertainty quantification. *Sci Data* 2021;**8**:1–17. https://doi.org/10.1038/s41597-021-00973-0.

2 Warren JL, Kong W, Luben TJ, Chang HH. Critical window variable selection: Estimating the impact of air pollution on very preterm birth. *Biostatistics* 2020;**21**:790–806. https://doi.org/10.1093/biostatistics/kxz006.

3 Warren J, Fuentes M, Herring A, Langlois P. Spatial-Temporal Modeling of the Association between Air Pollution Exposure and Preterm Birth: Identifying Critical Windows of Exposure. *Biometrics* 2012;**68**:1157–67. https://doi.org/10.1111/j.1541-0420.2012.01774.x.

4 George EI, McCulloch RE. Variable selection via Gibbs sampling. *J Am Stat Assoc* 1993;**88**:881–9. https://doi.org/10.1080/01621459.1993.10476353.
